# Macrophage exosomal ADAM10 mediates alveolar epithelial apoptosis induced by wood smoke PM_2.5_

**DOI:** 10.1007/s10565-026-10179-y

**Published:** 2026-03-29

**Authors:** Na Zhan, Yufeng Wang, Ru Liang, Yousen Wu, Ting Huang, Yuquan Ling, Xinbei Chen, Jiacheng Deng, LiFen Zhou, Kangni Luo, Yilin Cai, Yong-sheng Tu, Lihui Qu, Jianhua Li

**Affiliations:** 1 School of Basic Medical Sciences, Key Laboratory of Protein Modification and Degradation, State Key Laboratory of Respiratory Disease, Guangdong Basic Research Center of Excellence for Respiratory Medicine, Guangzhou Medical University, Guangzhou, Guangdong 511436 R.P. China; 2 KingMed College of Laboratory Medicine, Guangzhou Medical University, Guangzhou, Guangdong 511436 R.P. China; 3 The Fourth Affiliated Hospital of Guangzhou Medical University, Guangzhou Medical University, Guangzhou, 511300 R.P. China

**Keywords:** Emphysema, Wood smoke–derived PM_2.5_, Exosomes, ADAM10, SNAP23

## Abstract

Wood smoke (WS)-derived PM_2.5_ is a major environmental risk factor for emphysema, but the role of macrophage-derived exosomes remains unclear. Here, we show that chronic WS exposure in rats induces emphysematous lesions accompanied by markedly increased exosome release, reflected by elevated CD63 expression in alveoli and increased exosome particle number in bronchoalveolar lavage fluid. Pharmacological inhibition of exosome secretion with GW4869 significantly attenuated alveolar destruction. Moreover, intranasal administration of exosomes from PM_2.5_-exposed macrophages was sufficient to recapitulate emphysematous pathology *in vivo*. Mechanistically, PM_2.5_ exposure upregulated the membrane-trafficking protein SNAP23 in macrophages, enhancing exosome secretion and increasing total abundance of ADAM10 within exosomes. Co-localization of CD63 and ADAM10 was observed in alveolar regions of WS-exposed rats and COPD patient lungs. Silencing ADAM10 suppressed caspase-3 activation and reduced epithelial apoptosis, supporting a role for exosomal ADAM10 in driving caspase-3–dependent alveolar epithelial injury. By defining an exosome-mediated macrophage–epithelium pathway that contributes to PM_2.5_-induced emphysema, this study clarifies how combustion-derived PM_2.5_ damages lung structure and offers mechanistic insight to reducing the health burden of biomass-related air pollution.

**Graphical Abstract:**

1. Chronic wood smoke exposure increases exosome release in rat lungs.

2. PM_2.5_ upregulates SNAP23 in macrophages, promoting exosome secretion and ADAM10 enrichment.

3. Macrophage exosomal ADAM10 induces alveolar epithelial apoptosis via caspase-3 activation.

4. Targeting exosome release or ADAM10 offer potential strategies for PM_2.5_-induced emphysema.

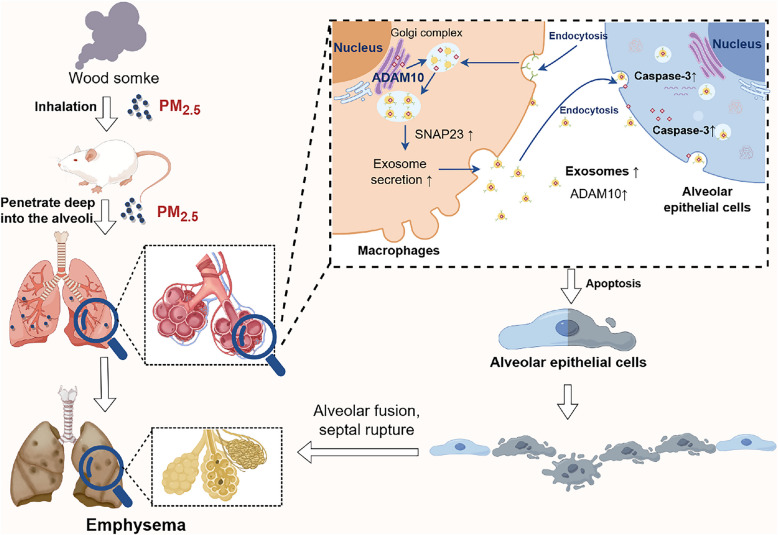

**Supplementary Information:**

The online version contains supplementary material available at 10.1007/s10565-026-10179-y.

## Introduction

Biomass burning, including residential wood combustion, wildfires, and prescribed fires, constitutes one of the major global sources of fine particulate matter (PM_2.5_), contributing substantially to both indoor and outdoor air pollution (Sun et al. 2019; Xu et al. 2020a, b; WHO 2024). Wood smoke (WS), a dominant component of emissions from these combustion processes (Ford et al. 2018; Olsen et al. 2020, Agency 2021), is a complex mixture containing at least 26 compounds designated by the U.S. Environmental Protection Agency as hazardous air pollutants, along with multiple known human carcinogens (Pye et al. 2024; Krasovich Southworth et al. 2025). Among these components, PM_2.5_ is widely recognized as the principal indicator of exposure and the key driver of smoke-related toxicity (Agency 2021; Reid et al. 2016; Rice et al. 2025). The small aerodynamic diameter of PM2.5 enables it to penetrate deep into the alveoli, where it triggers inflammation and tissue injury, establishing a strong link to the pathogenesis and progression of chronic obstructive pulmonary disease (COPD) (Hou et al. 2020; Li et al. 2023; Zhao et al. 2019). WS-derived PM_2.5_ is more toxic than other sources, impairing lung function in healthy individuals and exacerbating COPD symptoms (Aguilera et al. 2021; Schwartz et al. 2020; Olsen et al. 2020; Orru et al. 2022; Leng et al. 2022; Adeloye et al. 2022).

Emphysema, defined by irreversible alveolar wall destruction and airspace enlargement, is a major pathological component of COPD (Li et al. 2023; Zhao et al. 2019). Multiple epidemiological and experimental studies have linked emphysema progression to PM_2.5_ exposure (Xu et al. 2020a, b; He et al. 2017; Mohajeri et al. 2023; Yang et al. 2021), yet the cellular and molecular mechanisms that translate particulate exposure into structural tissue damage remain incompletely defined. Increasing attention has focused on extracellular vesicles (EVs), particularly exosomes, as mediators of intercellular communication in chronic lung diseases (Maas et al. 2017; Genschmer et al. 2019; Wu et al. 2023). Exosomes can transfer diverse cargos, including proteins, lipids, and RNAs, thereby modulating inflammation, epithelial injury, and tissue remodeling (Maas et al. 2017; Genschmer et al. 2019). Environmental particulates, such as PM_2.5_, have been shown to alter exosome release and cargo composition in airway and immune cells (Meng et al. 2022), suggesting that EV-mediated signaling may be an important link between particulate exposure and downstream tissue responses. However, the upstream molecular machinery that governs PM_2.5_-induced exosome secretion**,** as well as the specific exosomal cargo responsible for epithelial injury, remain poorly defined.

Protease-driven disruption of epithelial homeostasis is a central event in the pathogenesis of emphysematous injury. While matrix metalloproteinases have long been recognized for their roles in extracellular matrix turnover (Shimoda and Khokha 2017b; Genschmer et al. 2019), membrane-anchored sheddases provide an additional and less explored regulatory layer at the cell surface. The A Disintegrin and Metalloprotease (ADAM) family comprises type I transmembrane zinc-dependent proteases with modular architecture—including pro-domain, metalloprotease, disintegrin, cysteine-rich, and cytoplasmic regions—that collectively enable substrate recognition and catalytic activation (Zong et al. 2016; Shimoda and Khokha 2017a; Zheng et al. 2020; Werny et al. 2023). Many ADAMs function as sheddases that release bioactive ectodomains of cytokines, adhesion molecules, and growth-factor precursors, thereby rapidly reshaping epithelial–immune communication under inflammatory stress (Wang et al. 2023). Although ADAM-mediated proteolysis has been implicated in structural lung remodeling (Saitoh et al. 2009), how environmental particulates regulate ADAM family activation, trafficking, or incorporation into EVs remains largely unknown. Given that both latent and mature ADAM isoforms can be detected within exosomes (Shimoda and Khokha 2017b), EV-associated metalloproteases represent a plausible but understudied mechanism linking particulate exposure to epithelial injury. However, whether PM_2.5_ actively promotes the packaging of specific ADAM family members into exosomes, and how this process is regulated at the level of exosome biogenesis and secretion, have not been characterized.

Here, we investigated the role of macrophage-derived exosomes in mediating alveolar epithelial injury following exposure to WS-derived PM_2.5_. Our results show that PM_2.5_ exposure upregulates the SNARE protein SNAP23 in macrophages, thereby enhancing exosome secretion. This SNAP23-dependent increase in exosome release leads to elevated production and delivery of ADAM10-enriched exosomes to alveolar epithelial cells. Once internalized, these exosomes induce epithelial apoptosis in a caspase-3-dependent manner. Collectively, our findings identify a non-cell-autonomous mechanism whereby macrophages serve as signal amplifiers that propagate PM_2.5_-induced injury via SNAP23-regulated exosome secretion and ADAM10-mediated epithelial apoptosis. Our findings provide new insight into exosome-mediated crosstalk in emphysema and deepen our understanding of the molecular mechanisms underlying PM2.5-induced lung injury, highlighting SNAP23 and ADAM10 as potential therapeutic targets and offering valuable implications for environmental risk assessment and the management of emerging PM_2.5_-related health hazards.

## Materials and methods

### PM_2.5_ extraction

PM_2.5_ was provided by Professor Ran Pixin (Guangzhou Medical University, China), with purity reaching 99.8%, as verified in previous research (Huang et al. 2017). The PM_2.5_ extraction and purification process is briefly outlined below: PM_2.5_ was collected from the combustion of Chinese fir wood using a high-volume sampler (TE-6070, Tisch, USA), fitted with a PM_2.5_-selective inlet head (1.13 m^3^/min) (Ye et al. 2018; Gao et al. 2022). Particles were captured on fiberglass filters (1.6 μm pore size, 406 cm^2^ area), then dissolved in DMSO, filtered (5 μm), and stored as a 1 g/mL stock solution at −80 °C.

### Cell culture

Both the RAW 264.7 macrophage (JNO-RAW264, Jennio) and mouse lung epithelial-12 (MLE-12, CM3072, Xin Run Biotechnology) cell lines were cultured in their respective basal media, DMEM for the former and DMEM/F12 for the latter, each supplemented with 10% FBS and 1% penicillin–streptomycin (15140–122, Gibco). Both cell lines were cultured under standard conditions (37 °C, 5% CO₂, humidified atmosphere) and subsequently utilized for exosome-related experiments.

### Purification of the exosomes

RAW 264.7 cells were seeded in 150 mm cell culture dishes and treated with 15 μg/mL PM_2.5_ for 48 h. Cell culture supernatants were then collected, and exosomes were purified using differential velocity centrifugation. The medium was centrifuged at 500 × g for 10 min at 4 °C (Thermo Fisher, SL16R) to remove dead cells and large cell debris, followed by centrifugation at 2,000 × g for 10 min at 4 °C to remove small cell debris. Subsequently, supernatants were centrifuged at 10,000 × g for 30 min and filtered through a 0.22 µm filter. Following ultracentrifugation at 150,000 × g for 1.5 h at 4 °C (Beckman Coulter Optima XPN-100), exosome pellets were resuspended in cold PBS and stored at − 80 °C.

### Exosome characterization

The morphology of isolated exosomes was visualized with transmission electron microscopy (JEM-1400 PLUS, JEOL, Japan). Exosome samples (5 μL) were diluted with PBS, applied to copper grids, stained with 2% uranyl acetate, air-dried, and imaged at 80 kV.

Exosome samples were diluted in PBS to an optimal particle concentration and gently mixed to avoid vesicle rupture. Their size distribution and concentration were then analyzed using nanoparticle tracking analysis. Measurements were performed under standard settings (sensitivity: 70; shutter: 70), and samples were analyzed using a calibrated instrument. Data were considered valid when > 95% of particles ranged between 30–150 nm with a unimodal distribution.

### Animals

Eight-week-old male Sprague–Dawley (SD) rats were obtained from Guangzhou Ruige Biological Technology Co., Ltd. (Guangzhou, China). The rats were housed in a specific pathogen-free environment at 22 ± 2˚C, with a relative humidity of 50 ± 5%, and provided with standard animal diet and water available ad libitum. All animal procedures were approved by the Ethics Committee of the Laboratory Animal Center of Guangzhou Medical University (approval number: GY2022-103). The animals were housed at the animal facility of Guangzhou Medical University, and humane endpoints were implemented to minimize suffering.

### Animal experimental procedures

#### WS exposure

SD rats were randomly divided into six groups (n = 12 per group): three control groups exposed to filtered air for 1, 2, and 4 months, and three experimental groups exposed to PM_2.5_-rich WS for the same durations. WS was generated by the smoldering of Chinese fir sawdust in a controlled combustion chamber. Each exposure session lasted for 3 h and was conducted twice daily, 6 days per week, using a whole-body exposure system designed to simulate realistic indoor biomass burning conditions.

WS was generated in a wood-burning chamber and delivered at 5 L/min via a piston pump into a sealed, Teflon-coated animal exposure room, where fresh air was simultaneously introduced at 2.5 L/min to maintain circulation. An internal fan ensured homogeneous particle distribution, and pressure differentials (~ 5 Pa) between chambers ensured unidirectional smoke flow. Environmental parameters, including PM_2.5_ concentrations and gaseous components, were monitored through designated sampling ports throughout the exposure period (He et al. 2017).

The exposure concentration for rats in our study was set at 15 mg/m^3^ for 6 h per day, resulting in a cumulative daily exposure dose of 90 mg·h/m^3^. This dose was chosen based on prior pilot data indicating that it does not induce acute toxicity or mortality in rats (He et al. 2017). By comparison, typical human indoor PM_2.5_ exposure in rural households using traditional biomass stoves averages approximately 0.408 mg/m^3^ over a 24-h period (Mohajeri et al. 2023), corresponding to a cumulative daily exposure of about 9.8 mg·h/m^3^ (Table S1). Although the rat cumulative exposure dose appears higher, it is important to consider species-specific respiratory physiology and dosimetric differences when comparing animal exposure regimes to human real-world exposures. Rat inhalation exposures are frequently designed with shorter duration and higher concentrations to approximate chronic low-level exposures in a feasible laboratory timeframe. This approach is consistent with established inhalation dosimetry principles, which account for differences in exposure duration, respiratory tract deposition, and interspecies physiology when extrapolating between experimental models and human exposures (Oller and Oberdörster 2016; Agency 1994).

#### GW4869 treatment

SD rats were randomly allocated to five experimental groups (n = 9 per group): untreated control, solvent control (DMSO), GW4869 treatment, WS-exposed solvent, and WS-exposed GW4869 treatment. The exosome inhibitor GW4869 (0.5 mg/mL, Med Chem Express, USA) was delivered by tracheal instillation (200 μL) once weekly for 4 months, with corresponding vehicle (DMSO) administered to the solvent groups.

#### Exosome distribution and localization

To assess the *in vivo* distribution of exosomes, SD rats were randomly assigned to three groups (*n* = 12 per group): (1) a control group receiving intratracheal instillation of 200 μL PBS; (2) an exosome control group receiving 200 μL of PKH67-labeled exosomes derived from PBS-stimulated macrophages (100 μg/mL); (3) a PM_2.5_-exosome group receiving 200 μL of PKH67-labeled exosomes derived from PM_2.5_-stimulated macrophages (100 μg/mL). Exosomes were prepared as described in Section "Purification of the exosomes". Briefly, RAW 264.7 macrophages were exposed to 15 μg/mL PM_2.5_ or PBS for 24 h, and exosomes were isolated by differential ultracentrifugation. For fluorescent labeling, purified exosomes were incubated with PKH67 dye according to the manufacturer's protocol. Labeled exosomes were then washed and resuspended in PBS for instillation. Instillations were performed every other day for either 14 days or 1 month, depending on the experimental schedule.

#### Airway instillation of exosomes

SD rats were randomly assigned to three groups (n = 6 each) and received intratracheal instillations every other day for 1 or 2 months: PBS control, exosome control (PBS-stimulated macrophage-derived exosomes, 100 μg/mL), or PM_2.5_-exosome (PM_2.5_-stimulated macrophage-derived exosomes, 100 μg/mL). Exosomes were prepared according to the procedure described in the section "Purification of the exosomes". Each rat received an intratracheal instillation of 200 μL of exosome suspension (containing 20 μg of total exosomal protein) under light anesthesia. Instillations were carried out every other day for either 1 month or 2 months. This protocol was designed to mimic prolonged pulmonary exposure to macrophage-derived exosomes following PM_2.5_ stimulation.

### Cell viability assay

Exosomes used for cell treatment were quantified by total protein content as described in Section "Exosome characterization". MLE-12 cells (2 × 10^4^ cells/well) were seeded in a 96-well plate and treated with exosomes secreted by RAW 264.7 macrophages that had been stimulated with varying doses of PM_2.5_ (0, 25, 50, 100, 150 µg/mL) for 24 h. Cell viability was assessed using the CCK-8 kit (Beyotime, China) according to the manufacturer's instructions.

### Lentiviral transfection

RAW 264.7 cells were transduced with lentiviruses encoding shRNA targeting SNAP23 or ADAM10 (GeneChem, Shanghai, China). Lentiviruses carrying a non-targeting shRNA were used as a control. Cells were infected at a multiplicity of infection (MOI) of 80 in the presence of 8 µg/mL polybrene (Sigma-Aldrich) and incubated for 24 h. After 48 h of infection, the culture medium was replaced, and stable transductants were selected using 2 µg/mL puromycin for 5 days. Transduction efficiency was assessed by GFP fluorescence under a fluorescence microscope (Leica DMi8) and verified by quantitative PCR and Western blotting.

### Co-culture assay

In the co-culture system, the lower chambers of transwell plates were seeded with MLE-12 cells (5.0 × 10^5^ cells/well) in DMEM/F12 supplemented with 10% FBS. The inserts were subsequently populated with RAW 264.7 cells (5.0 × 10^4^ cells/well) cultured in DMEM with 10% FBS. After 24 h, the medium was replaced with serum-free DMEM, and RAW 264.7 cells were treated with either DMSO or the exosome inhibitor GW4869 (Med Chem Express, USA). Co-cultures were maintained for 48 h, after which MLE-12 cells were collected in the lower chamber.

### Immunofluorescence staining

**Cell-based IF**: To evaluate E-cadherin expression and exosome uptake, MLE-12 cells were incubated with PKH67-labeled exosomes (MINI67, Sigma-Aldrich) for 2 or 24 h. Cells on coverslips were fixed with 4% paraformaldehyde (PFA), permeabilized with 0.1% Triton X-100, and blocked with goat serum. Primary antibody against E-cadherin (PB9561, Boster), CD63 (10628D, Thermo Fisher) was applied overnight at 4 °C, followed by a fluorescent secondary antibody (550,075, Zenbio, China; 1:500) for 1 h. Nuclei were stained with DAPI (C1005, Beyotime, China). Images were acquired using a Zeiss LSM880 confocal microscope.

**Rat lung tissues:** WS-exposed rat lung sections were fixed (4% PFA), permeabilized (0.1% Triton X-100), and blocked (5% BSA). Primary antibody incubation (anti-CD63, 10628D, Thermo Fisher; anti-ADAM10, ab1997, Abcam) was carried out overnight at 4 °C, followed by Alexa Fluor 488 and 594 secondary antibodies. Nuclear counterstaining was achieved with DAPI.

**Human COPD tissues:** Formalin-fixed, paraffin-embedded lung tissue specimens were obtained from the First Affiliated Hospital of Guangzhou Medical University, with kind support from Professor Wenju Lu. The COPD group consisted of two males and one female, aged 56–77 years, with GOLD stages 1–2, and included both smokers and non-smokers. The control group comprised two males and one female aged 50–73 years, with no history of respiratory disease and normal pulmonary function (Table S2). All COPD patients included in this study had a confirmed diagnosis of emphysema based on both pulmonary function tests (post-bronchodilator FEV1/FVC < 0.70, GOLD criteria) and high-resolution computed tomography (HRCT) imaging demonstrating characteristic emphysematous changes. All samples were obtained from patients undergoing lung resection for unrelated clinical reasons at the First Affiliated Hospital of Guangzhou Medical University. Ethical approval was granted by the Ethics Committee of the First Affiliated Hospital of Guangzhou Medical University (Approval No.ES-2024–030-02). Clinical trial number: not applicable. All participants or their legal representatives provided written informed consent before sample collection, following the principles of the Declaration of Helsinki.

Tissue sections underwent antigen retrieval (10 mM citrate buffer, 95 °C, 20 min), followed by blocking and antibody incubation steps identical to those used for rat tissues. Specificity was confirmed using isotype control antibodies. All images were acquired using a Zeiss LSM880 confocal microscope with a 20 × objective lens. Co-localization was quantified using the JACoB plugin in ImageJ and expressed as Manders’ overlap coefficient.

### Apoptosis

Apoptosis in MLE-12 cells was detected using flow cytometry and immunofluorescence microscopy. Cells were stained with Annexin V/PI (KGA107, Keygen) per manufacturer's protocol: after washing and digestion, cells were resuspended in PBS, incubated with 5 µL each of Annexin V and PI for 10 min at room temperature in the dark, and then analyzed by confocal microscopy (ZEISS LSM880) and flow cytometry (BD; FlowJo software.

### Immunoelectron microscope

Purified exosomes suspended in PBS were deposited onto the carbon-coated grids. The grids were incubated with an anti-ADAM10 antibody (ab125011, Abcam) for 30 min at room temperature, followed by incubation with a goat anti-rabbit IgG H&L antibody (bs-0295G-Gold, Bioss) for an additional 30 min. Negative staining was performed using 2% phosphotungstic acid hydrate (Aladdin). Grids were air-dried and visualized using a JEM-1400 PLUS transmission electron microscope.

### Western blot

Exosomes were collected from equal numbers of cells, and protein loading was normalized based on total protein concentrations determined from corresponding cell lysates. Proteins were separated by 12% SDS-PAGE and transferred onto PVDF membranes (Millipore, USA). Membranes were blocked and incubated with primary antibodies against CD9 (ab92726, Abcam), TSG101 (ab125011, Abcam), CD68 (ab125212, Abcam), E-cadherin (PB9561, Boster), ADAM10 (ab124695, Abcam), cleaved caspase-3 (82707–13-RR, proteintech), BCL2 (82469–6-RR, proteintech), and GAPDH (HRP-60004, Proteintech) overnight at 4 °C. After washing, membranes were incubated with HRP-conjugated secondary antibodies (BF03001, Bioword), followed by ECL detection and ImageJ quantification of protein bands. Signal intensities were normalized to GAPDH.

### RNA preparation and qPCR

Total RNA from macrophages infected with or without *shAdam10* lentivirus was extracted using the Steadypure universal RNA extraction kit following the manufacturer's instructions (15596,018, Hunan Eckeri Bioengineering Co., LTD.). QPCR was performed to detect *Adam10* relative expression and normalized to *Gapdh* as a housekeeping gene. Relative RNA expression was calculated by ΔΔCt method. Primers for qPCR were as follows.

Mouse *Adam10* primer:

Forward: 5 '-ATGGTGTTGCCGACAGTGTTA-3'.

Reverse: 5 '-GTTTGGCACGCTGGTGTTTTT-3'.

### Pulmonary morphometric and pathology assessment

Following pulmonary function testing, rats were euthanized, and the trachea was cannulated with ligation of the left main bronchus. Bronchoalveolar lavage of the right lung was performed using three 0.8 mL PBS instillations; the first BALF aliquot was centrifuged and the supernatants stored at − 80 °C. The right lung was subsequently excised and frozen at − 80 °C, while the left lung was fixed in 4% PFA, paraffin-embedded, and stained with hematoxylin and eosin (HE). The mean linear intercept (MLI) was used to assess lung morphometry. Briefly, a cross-shaped reticle was centered in the visual field, and alveoli intersecting the reticle lines were counted. MLI was calculated as the total reticle length divided by the number of intersecting alveoli.

### Extraction of exosomes from BALF

BALF pooled from six rats was subjected to sequential centrifugation (500 × g, 2000 × g, 10,000 × g) and filtration, followed by ultracentrifugation at 150,000 × g for 1.5 h at 4 °C (Beckman Coulter, Coulter Optima XPN-100). The exosome pellet was resuspended in PBS and stored at −80 °C.

### ADAM10 expression in COPD using GEO Datasets

The expression profile of ADAM10 in COPD was investigated using the publicly available RNA-seq dataset GSE57148 from the Gene Expression Omnibus (GEO) database (https://www.ncbi.nlm.nih.gov/geo/query/acc.cgi?acc=GSE57148). The dataset comprises 98 COPD patients and 91 healthy controls. As described by the original submitters, the dataset was generated through the following pipeline: RNA-seq reads were aligned to the human reference genome (hg19) using TopHat (v1.4.1), and gene-level expression quantification was performed with Cufflinks software (v2.0.0) to generate Fragments Per Kilobase of transcript per Million mapped reads (FPKM) values. The preprocessed data underwent quality control measures including: (i) elimination of genes exhibiting zero FPKM values across all samples, and (ii) upper quantile normalization to correct for technical variation. For our analysis, we directly utilized the normalized FPKM expression matrix (genes × samples) provided in the supplementary files. Differential expression assessment of ADAM10 between COPD and control groups was performed on these preprocessed data.

### Single-cell transcriptomic analysis of ADAM10 in lung

Publicly available single-cell RNA sequencing data from the Human Protein Atlas (HPA, version 21.0, https://www.proteinatlas.org) were used to assess ADAM10 expression in human lung cell populations. The UMAP (Uniform Manifold Approximation and Projection) plots showing ADAM10 expression across annotated lung cell populations were retrieved directly from the HPA database under the "SINGLE CELL TYPE" section for lung tissue (https://www.proteinatlas.org/ENSG00000137845-ADAM10/single+cell/lung).

### Statistical analysis

GraphPad Prism 9 was used for statistical analysis. The Shapiro–Wilk test was applied to assess data normality. For parametric data, results are expressed as mean ± standard deviation (SD) and comparisons were made using Student's t-test or ANOVA with Tukey's post hoc test; otherwise, the Mann–Whitney U test was employed.

## Results

### Exosomes contribute to WS-induced emphysema

**Fig. 1:**
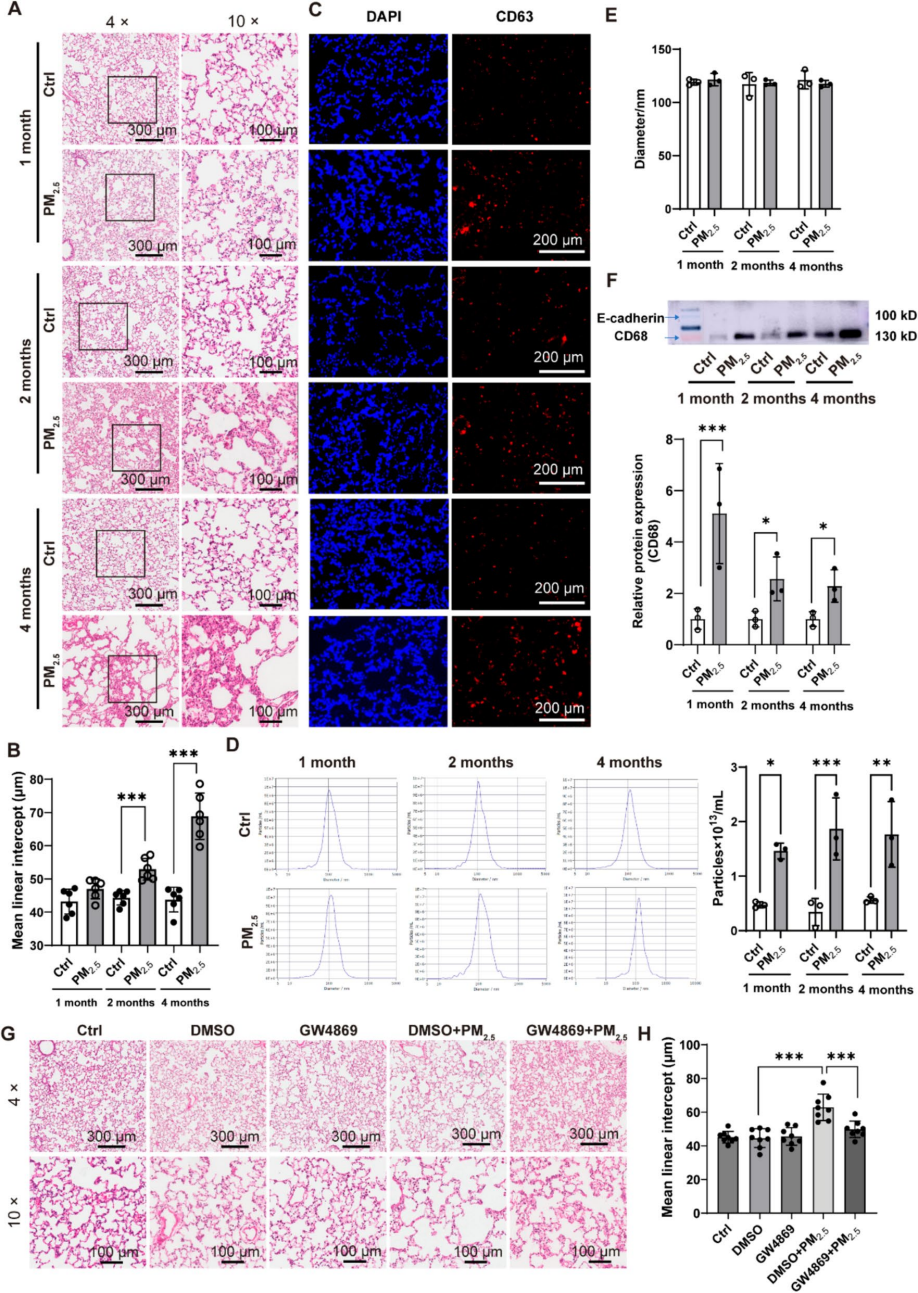
Exosomes contribute to wood smoke (WS)-induced emphysema in rats. SD rats were exposed to PM_2.5_-rich WS for 1, 2, or 4 months (*n* = 6). (**A**) Representative H&E-stained lung sections. (**B**) Quantification of mean linear intercept (MLI) (*n* = 6). (**C**) Immunofluorescence of CD63^+^ exosomes (red) and nuclei (DAPI, blue). (**D**, **E**) Nanoparticle tracking analysis (NTA) of bronchoalveolar lavage fluid (BALF)-derived exosmoes showing the particle size distribution (left) and concentration (right) (D), and mean particle diameter (E). (**F**) Western blot analysis of CD68 and E-cadherin expression in BALF-derived exosomes, normalized to total exosomal protein. (**G**, **H**) H&E staining and MLI quantification after 4-month WS exposure with/without GW4869 (*n* = 9). Groups: Ctrl, clean air; PM_2.5_, WS-exposed; DMSO, vehicle; GW4869, inhibitor; combinations as indicated. Data are presented as mean ± SD. Statistical analysis was performed using unpaired two-tailed t-tests; ns, not significant; *p < 0.05, **p < 0.01, ***p < 0.001

Rats were exposed to PM_2.5_-rich WS for 1, 2, and 4 months to assess structural changes in the lung. After 1 month of exposure, no overt abnormalities were detected by histological examination. By 2 months, lungs exhibited features consistent with early emphysematous remodeling, including increased alveolar fusion, enlarged mean linear intercept (MLI), and localized septal rupture (Fig. 1A, B). These pathological changes worsened after 4 months. An increase in mononuclear cells within alveolar septa was also observed over time (Fig. 1A).

Immunofluorescence staining of lung tissue revealed increased CD63-positive exosomes over time (Fig. 1C), and NTA of BALF-derived exosomes revealed progressively higher numbers of extracellular vesicles within the exosomal size range (100-120 nm) without altering particle size distribution (Fig. 1D, E). Because exosomes lack stable housekeeping proteins for normalization, CD68 and E-cadherin levels were assessed under equal total exosomal protein loading. Under these conditions, expression of the macrophage marker CD68 increased across the exposure duration, whereas exosomal E-cadherin was barely detectable across all time points (Fig. 1F). The concomitant increase in CD63 signal and macrophage-associated exosomal CD68 indicates that macrophages may contribute to the elevated vesicle pool, although involvement of other cell types cannot be excluded.

To assess whether exosome-dependent processes contribute to WS-associated structural alterations, rats were treated with the neutral sphingomyelinase inhibitor GW4869 throughout the 4-month exposure period. GW4869 treatment reduced exosome numbers in BALF and was associated with attenuated structural abnormalities, including decreased alveolar fusion, lower MLI, and more preserved septal architecture compared with WS exposure alone (Fig. 1G, H). These findings support the involvement of exosome-related pathways in the lung remodeling observed during chronic WS exposure.

### PM_2.5_-induced macrophage exosomes mediate emphysema-like pathology

**Fig. 2:**
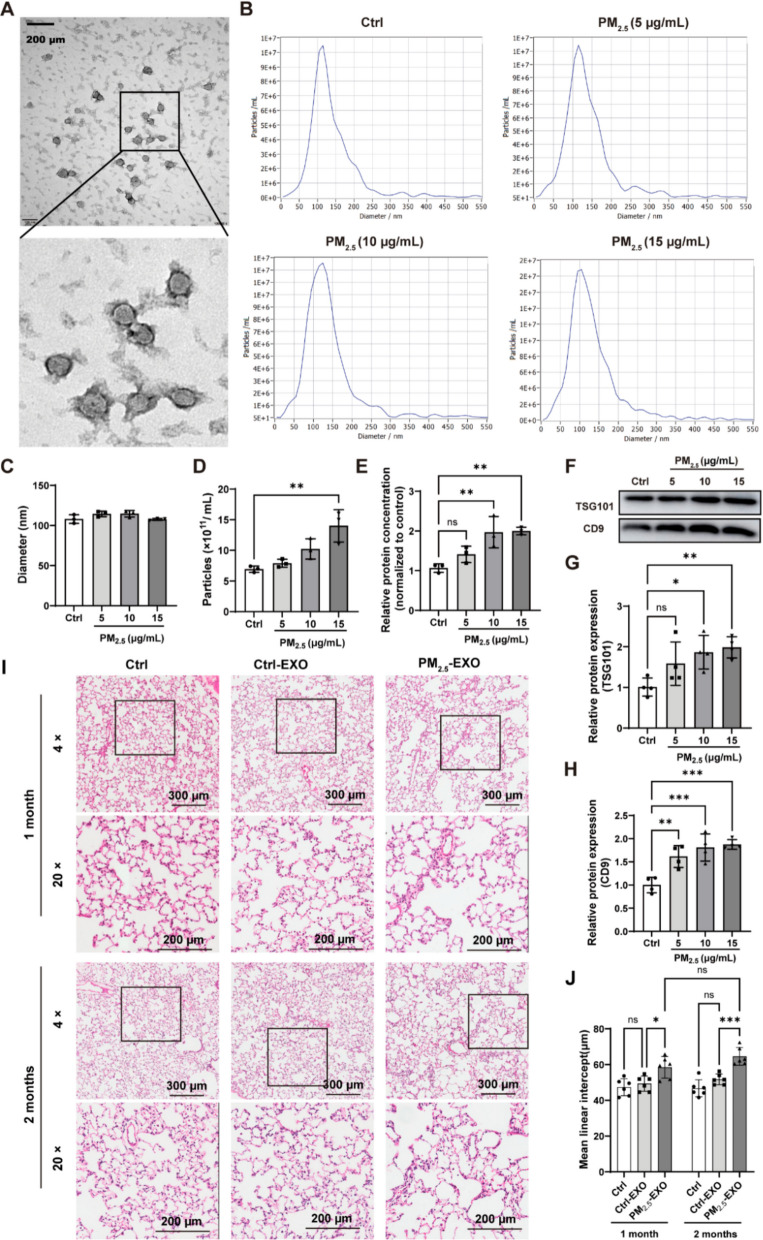
PM_2.5_ promotes macrophage exosome secretion and PM_2.5_-induced exosomes cause alveolar enlargement in vivo. (**A**) Transmission electron microscopy of exosomes from RAW 264.7 macrophages (upper panel: 50,000 ×; lower panel: 100,000 ×). Macrophages were exposed to 5, 10, or 15 μg/mL PM_2.5_, and exosomes were analyzed for: (**B**) particle size distribution, (**C**) mean particle diameter, (**D**) particle concentration, and (**E**) total protein content. (**F**–**H**) Western blot analysis of exosomal surface markers TSG101 and CD9, with quantification. (**I**) Representative H&E-stained lung sections from rats receiving macrophage-derived exosomes for 1 or 2 months. (**J**) MLI quantification (*n* = 6). Group: Ctrl, untreated control; Ctrl-EXO, exosomes from macrophages exposed to PBS; PM_2.5_-EXO, exosomes from macrophages exposed to PM_2.5_. Data are presented as mean ± SD. Statistical analysis were performed using one-way or two-way ANOVA as appropriate; ns, not significant; *p < 0.05, **p < 0.01, ***p < 0.001

Building on the *in vivo* correlation that WS exposure increased exosome release and that BALF exosomes showed elevated levels of the macrophage marker CD68 (Fig. 1F), we next examined key lung cell types in vitro to determine the cellular source and directly test the pathogenic role of these vesicles. This approach allowed us to quantify PM_2.5_-induced exosome secretion, characterize vesicle properties, and assess their capacity to induce alveolar injury. RAW264.7 macrophages and MLE-12 epithelial cells were exposed to PM_2.5_ in vitro. Vesicles isolated from RAW 264.7 displayed typical cup-shaped morphology (Fig. 2A) and a size centered around ~120 nm (Fig. 2B, C). PM_2.5_ exposure induced a dose-dependent increase in particle number and total vesicular protein in macrophages (Fig. 2D, E). Western blot analysis showed increased CD9 at all doses and increased TSG101 at higher doses (Fig. 2F–H). Under identical conditions, vesicle output from MLE-12 cells was near the detection limit (Supplementary S1), and thus no interpretable TEM images could be obtained. These findings identify macrophages as a substantial source of PM_2.5_-responsive vesicles, though other lung cell types may also contribute.

To evaluate their functional relevance, exosomes isolated from PM_2.5_-treated macrophages (PM_2.5_-EXO) were administered intratracheally to rats. PKH67-labeled exosomes successfully localized to lung tissue (Fig. S1). Rats receiving PM_2.5_-EXO developed alveolar enlargement, septal rupture, and increased MLI after one month, whereas exosomes from PBS-exposed macrophages (Ctrl-EXO) produced milder changes (Fig. 2I, J). At 2 months, alveolar damage progressed, and MLI values trended higher than at 1 month (Fig. 2I, J), although without reaching statistical significance. These results indicate that increased macrophage-derived vesicles are sufficient to reproduce key features of WS-associated alveolar remodeling *in vivo.*

### Exosomes from PM_2.5_-stimulated macrophages induce alveolar epithelial cell apoptosis

Prompted by the *in vivo* evidence that macrophage-derived exosomes promote emphysema, we next examined whether these vesicles directly impair alveolar epithelial cells. Fluorescence microscopy revealed exosome internalization by MLE-12 as early as 2 h, with a dose- and time-dependent increase at 24 h (Fig. S2).

**Fig. 3:**
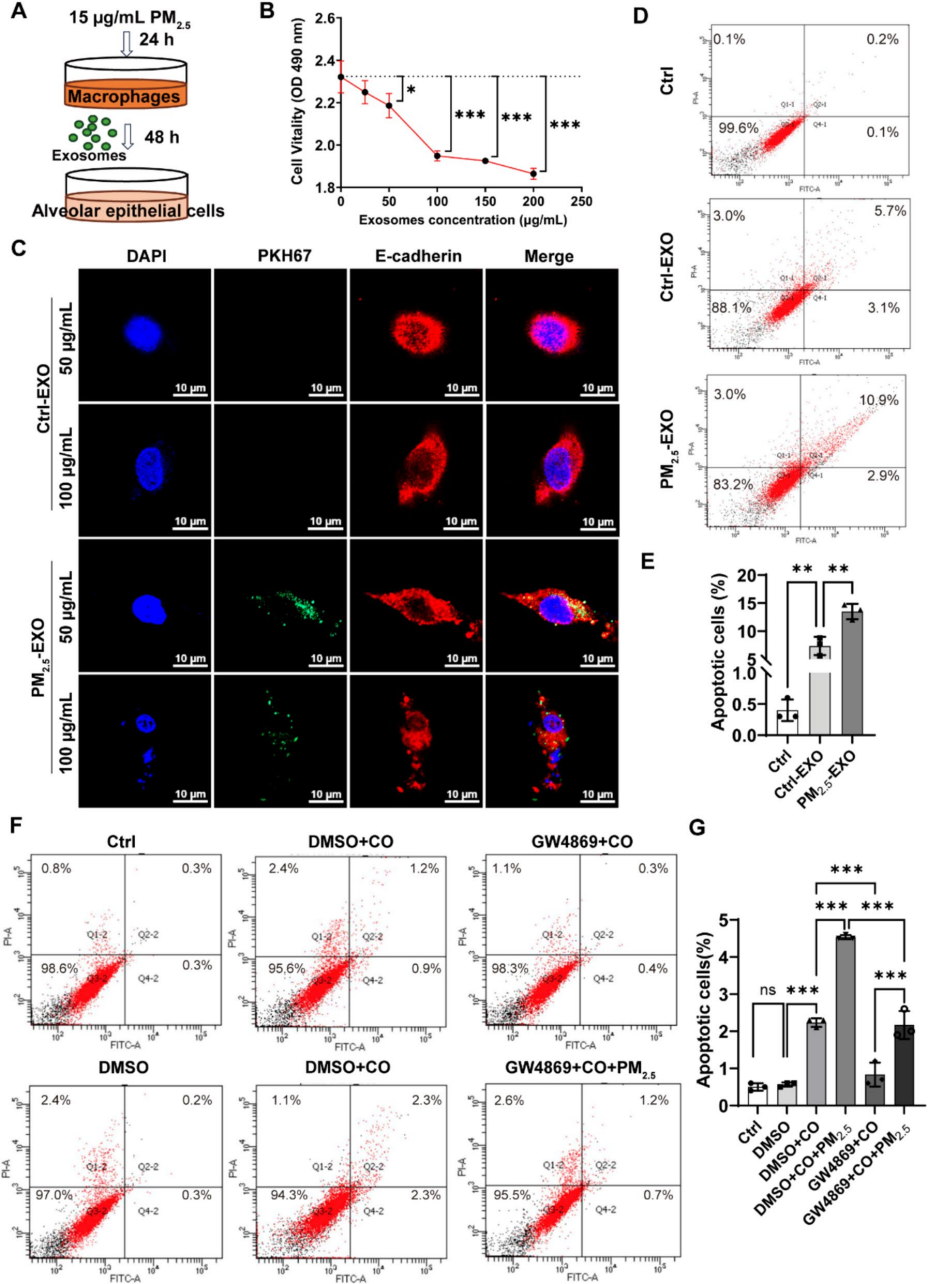
PM_2.5_-stimulated macrophage exosomes induce alveolar epithelial apoptosis. (**A**) Schematic of MLE-12 cells co-incubated with macrophage-derived exosomes. (**B**) Cell viability of MLE-12 cells after 24 h incubation with increasing concentrations (0, 25, 50, 100, 150, 200 µg/mL, quantified as total exosomal protein) of exosomes from PM_2.5_-treated macrophages. (**C**) Fluorescence microscopy of exosome uptake labeled with PKH67 (green); nuclei (DAPI, blue); and cell membranes stained with E-cadherin (red) ( scale bar = 10 μm). (**D**–**E**) Apoptosis of MLE-12 cells treated with 100 µg/mL PM_2.5_-EXO for 24 h, quantified as the sum of early and late apoptotic cells. (**F**) Effects of inhibiting macrophage exosome secretion (GW4869) on epithelial apoptosis, assessed by Annexin V-FITC/PI staining. Quadrants: viable cells (Q3), early apoptosis (Q4), late apoptosis (Q2), necrosis (Q1). (**G**) Quantification of apoptosis cells. Group: Ctrl, untreated control; Ctrl-EXO, exosomes from PBS-stimulated macrophages; PM_2.5_-EXO, exosomes from PM_2.5_-stimulated macrophages. DMSO, vehicle control; DMSO + CO, co-culture with DMSO-treated RAW 264.7 cells; GW4869 + CO, co-culture with GW4869-treated RAW 264.7 cells; DMSO + CO + PM_2.5_, co-culture with DMSO-treated RAW 264.7 cells under PM_2.5_ exposure; GW4869 + CO + PM_2.5_, co-culture of GW4869-treated RAW 264.7 cells under PM_2.5_ exposure. Data are presented as mean ± SD. Statistical analysis was performed using one-way ANOVA with Tukey’s post hoc test. Pairwise comparisons were performed using unpaired two-tailed Student’s t-test. Statistical significance is indicated as ns, not significant; **p < 0.01, ***p < 0.001

Cell viability analysis showed that PM_2.5_-EXO significantly reduced MLE-12 cell viability after 24 h, particularly at 50 μg/mL and higher concentrations (Fig. 3A, B). Morphological evaluation revealed nuclear shrinkage at 50 μg/mL and more pronounced chromatin condensation and fragmentation at 100 μg/mL, consistent with apoptotic features (Fig. 3C). Flow cytometry demonstrated higher apoptotic proportions in cells exposed to 100 μg/mL PM_2.5_-EXO compared with Ctrl-EXO, although Ctrl-EXO also induced mild apoptosis (Fig. 3D, E, Fig. S3).

A transwell macrophage–epithelium co-culture system further supported this observation. Co-culture with unstimulated macrophages significantly increased epithelial apoptosis compared with epithelial cells alone (Fig. 3F, G). PM_2.5_ exposure further amplified this apoptotic effect. GW4869 reduced apoptosis in both basal and PM_2.5_-stimulated conditions, but apoptosis in the PM_2.5_ + GW4869 group remained higher than with GW4869 alone (Fig. 3F, G). These results indicate that vesicle-dependent pathways contribute to epithelial apoptosis while also suggesting that PM_2.5_ may activate additional vesicle-independent injury mechanisms.

### PM_2.5_ activates the SNARE fusion machinery and requires SNAP23 for efficient vesicle release

**Fig. 4:**
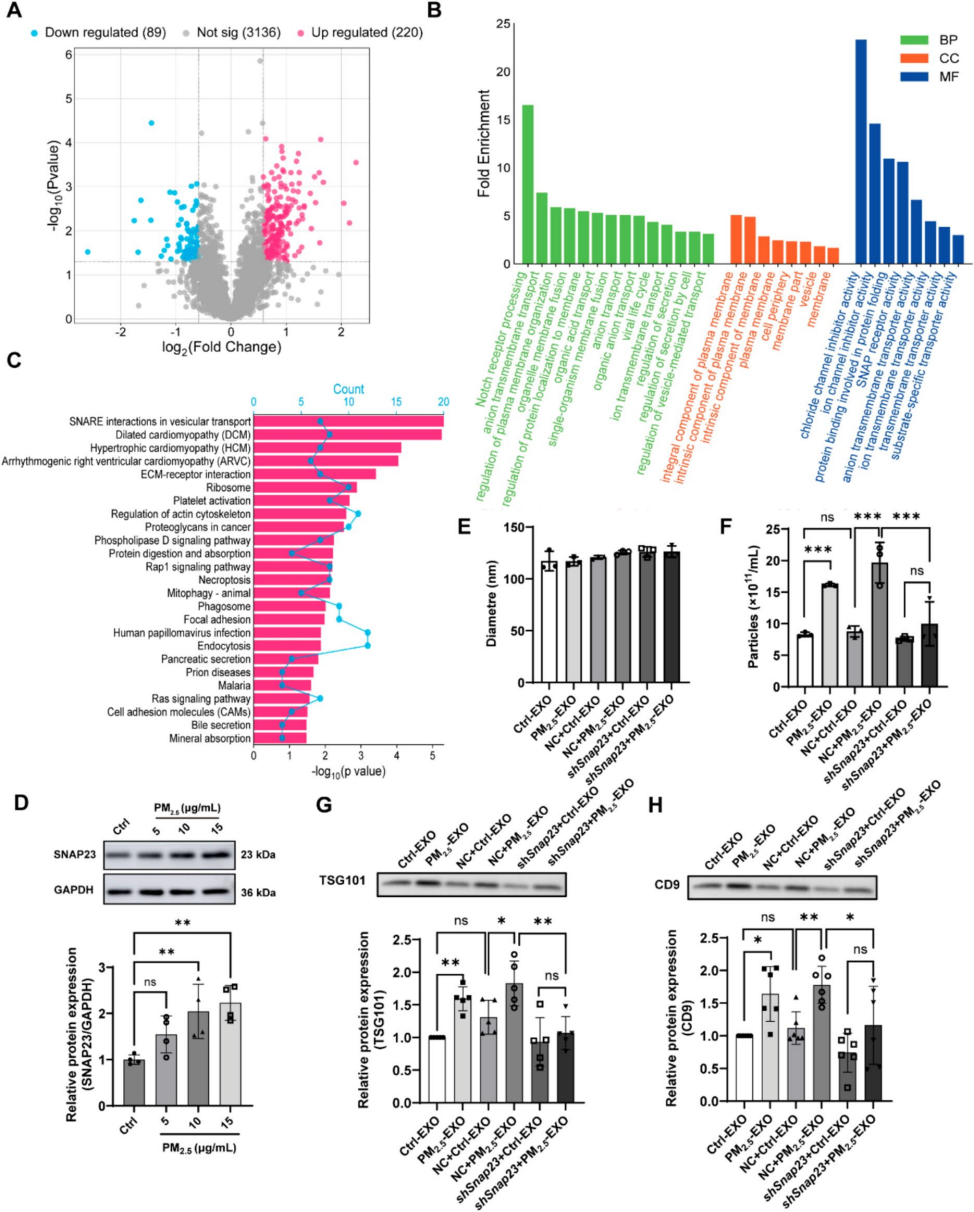
Proteomic analysis identifies SNAP23 as a key regulator of PM2.5-induced exosome release in macrophages. (**A**) Volcano plot of differentially expressed proteins (DEPs) in exosomes derived from PM_2.5_-stimulated macrophages compared with controls. (**B**) Gene Ontology (GO) enrichment analysis of upregulated DEPs. (**C**) Kyoto Encyclopedia of Genes and Genomes (KEGG) pathway analysis of upregulated DEPs. (**D**) SNAP23 expression in RAW 264.7 macrophages following 48-h stimulation with gradient concentrations of PM_2.5_; PBS-treated cells served as the 0 μg/mL control. (**E**–**F**) NTA of exosomes derived from *Snap23*-knockdown macrophages, including mean particle diameter (E) and particle concentration (F). (**G**–**H**) Western blot analysis and quantification of exosomal markers TSG101 (G) and CD9 (H) in exosomes from PM2.5-stimulated macrophages with or without Snap23 knockdown. Groups: Ctrl, exosomes from PBS-treated macrophages; PM_2.5_-EXO, exosomes from PM_2.5_-treated macrophages; NC, macrophages transduced with non-targeting control shRNA; sh*Snap23*, macrophages transduced with *Snap23*-targeting shRNA. Statistical analysis was performed using one-way ANOVA . or two-way ANOVA with Tukey’s post hoc test, as appropriate. Data are presented as mean ± SD; ns, not significant; *p < 0.05, **p < 0.01, ***p < 0.001

Quantitative proteomic profiling identified 309 differentially expressed proteins (DEPs) in PM_2.5_-EXO (220 upregulated, 89 downregulated; Fig. 4A). GO enrichment analysis of upregulated proteins showed overrepresentation of membrane-related cellular components, including “intrinsic component of membrane,” “plasma membrane,” “integral component of plasma membrane,” and “vesicle,” consistent with involvement in exosome biogenesis and trafficking (Fig. 4B). Enriched molecular functions such as “SNAP receptor activity,” and “transmembrane transporter activity,” together with biological processes including “regulation of secretion,” “vesicle-mediated transport,” and “organelle membrane fusion,” collectively pointed toward enhanced activity of the SNARE fusion machinery. KEGG analysis further supported this interpretation by highlighting enrichment of the “SNARE interactions in vesicular transport” pathway (Fig. 4C).

Among SNARE-associated proteins detected, multiple members—including Syntaxin family members (Stx4, Stx7, Stx8, Stx12), VAMP4, VAMP8, Vti1b, and SNAP23—showed moderate but coordinated increases (Table S3). The comparable magnitude of change across these components indicates that the proteomic signal reflects broad modulation of the SNARE apparatus, rather than selective upregulation of an individual factor.

To determine whether PM_2.5_ modulates the SNARE machinery at the cellular level, we examined the expression of SNAP23, a canonical t-SNARE that is required for plasma membrane fusion and has been previously implicated in exosome release(Wei et al. 2017). Western blot analysis showed a dose-dependent increase in SNAP23 expression in RAW264.7 macrophages following 48-h PM_2.5_ exposure, with a 2.23-fold elevation at 15 μg/mL relative to unstimulated cells (Fig. 4D). Silencing of *Snap23* using shRNA (sh*Snap23,* Fig. S4A) did not affect vesicle size distribution but markedly reduced particle number, exosomal protein content, and the levels of exosomal markers CD9 and TSG101 under PM_2.5_ stimulation (Fig. 4E–H). These findings indicate that SNAP23 is required for efficient exosome release in macrophages, consistent with its known role in SNARE-mediated membrane fusion.

### Macrophage exosomes regulate alveolar epithelial cell apoptosis by carrying ADAM10

**Fig. 5:**
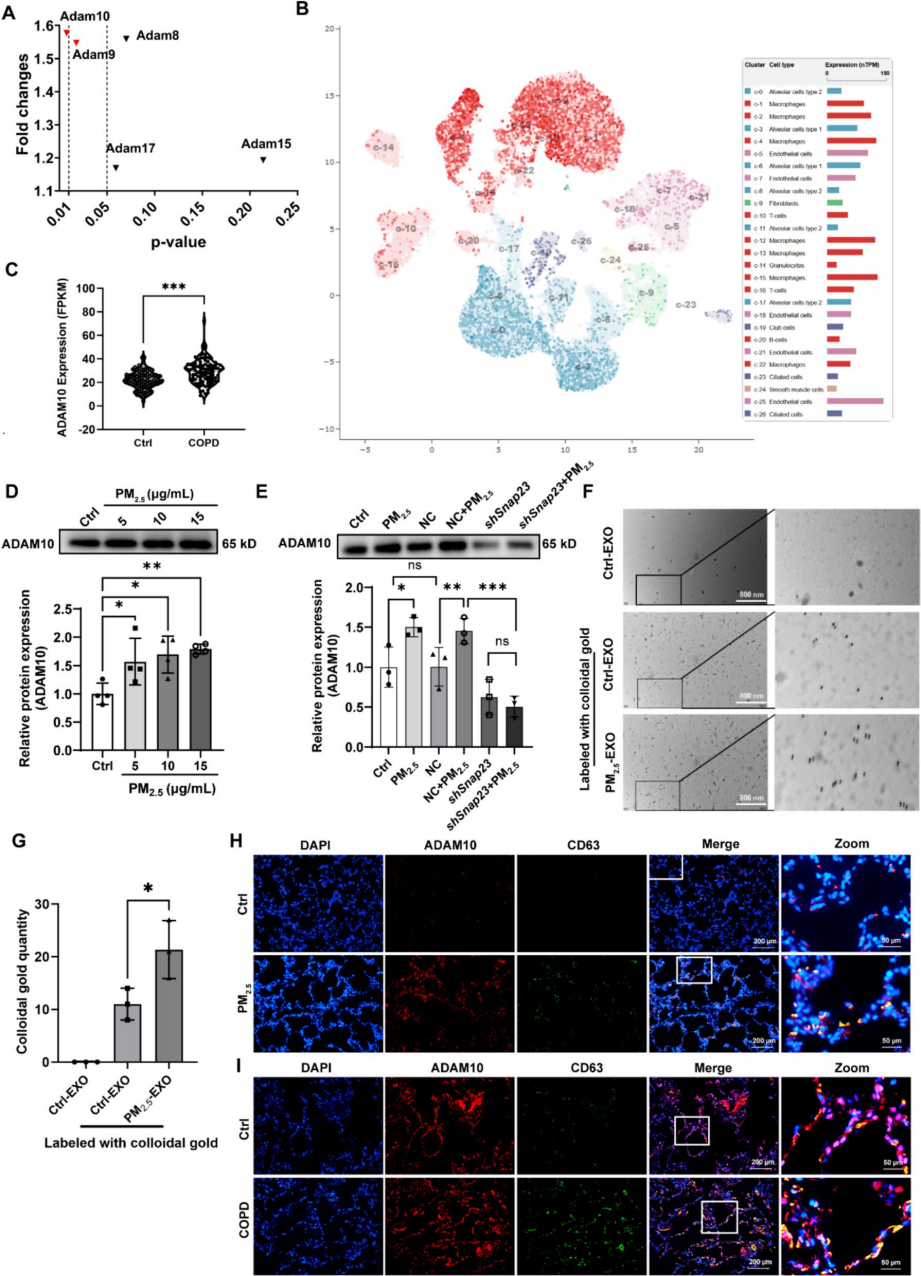
Macrophage exosomes regulate alveolar epithelial cell apoptosis through ADAM10. (**A**) Proteomic identification of ADAM family members in exosomes from PM_2.5_-treated macrophages. (**B**) Single-cell UMAP plot showing ADAM10 RNA expression across lung cell populations based on Human Protein Atlas (HPA, version 21.0, https://www.proteinatlas.org). Expression levels are shown as normalized transcript per million (nTPM). (**C**) ADAM10 mRNA expression in lung tissues from COPD patients (*n* = 98) and healthy controls (*n* = 91) using public RNA-seq dataset GSE57148 (available at https://www.ncbi.nlm.nih.gov/geo/query/acc.cgi?acc=GSE57148). Box plots represent the distribution of FPKM values after upper quantile normalization. (**D**) Protein expression level of ADAM10 in macrophages following PM_2.5_ exposure. (**E**) Effect of *Snap23* knockdown on ADAM10 protein expression in macrophages exposed to PM_2.5_. (**F**) Detection of ADAM10 expression and localization in macrophage-derived exosomes using colloidal gold-antibody labeling. Colloidal gold particles: 15 nm; scale bar: 800 nm; magnification: 50,000 ×. (**G**) Quantification of immungold particles per exosome (10 μg exosomes labeled; *n* = 3) after 48-h stimulation with 15 μg/mL PM_2.5_. (**H**, **I**) Immunofluorescence staining of rat lung sections after chronic WS exposure (H) and lung biopsies from COPD patients (I) (*n* = 3), showing CD63 (green), ADAM10 (red), and nuclei (DAPI, blue). Groups: Ctrl, untreated cells; PM_2.5_, exosomes from PM_2.5_-stimulated macrophages; NC, macrophages transduced with non-targeting shRNA; sh*Snap23*, macrophages transduced with *Snap23*-targeting shRNA. Statistical analysis was performed using one-way ANOVA or two-way ANOVA with Tukey’s post hoc test, as appropriate; pairwise comparisons were performed using unpaired two-tailed Student’s t-test. Data are presented as mean ± SD; ns, not significant; *p < 0.05, **p < 0.01, ***p < 0.001

Proteomic profiling showed that several ADAM family members—including ADAM8, ADAM9, ADAM10, ADAM15, and ADAM17—were enriched in PM_2.5_-EXO compared with Ctrl-EXO (Fig. 5A). Because ADAM proteins collectively participate in ectodomain shedding, epithelial barrier remodeling, and inflammatory signaling, we initially evaluated them as a functional group. Among these, ADAM10 exhibited the most consistent and statistically significant increase (fold change > 1.5, p < 0.01), motivating its further investigation.

Single-cell RNA-seq data from the Human Protein Atlas demonstrated that ADAM10 is expressed in human lung macrophages (Fig. 5B). An independent transcriptomic dataset (GEO: GSE57148) showed higher ADAM10 mRNA levels in COPD lungs compared with healthy controls (Fig. 5C), supporting potential disease relevance. Western blot analysis further confirmed that ADAM10 abundance in PM_2.5_-EXO increased in a dose-dependent manner (Fig. 5D).

Importantly, *Snap23* knockdown markedly reduced the PM_2.5_-associated increase in exosomal ADAM10 detected under equal-protein conditions (Fig. 5E), suggesting that SNAP23-dependent exosome release contributes to the elevated exosomal ADAM10 levels. Immunogold labeling detected ADAM10 on exosome surfaces and showed higher labeling density in PM_2.5_-EXO (Fig. 5F, G). Co-localization of CD63 and ADAM10 was observed along alveolar walls in WS-exposed rat lungs (Fig. 5H), and similar patterns were evident in human COPD lung tissue (Fig. 5I).

**Fig. 6:**
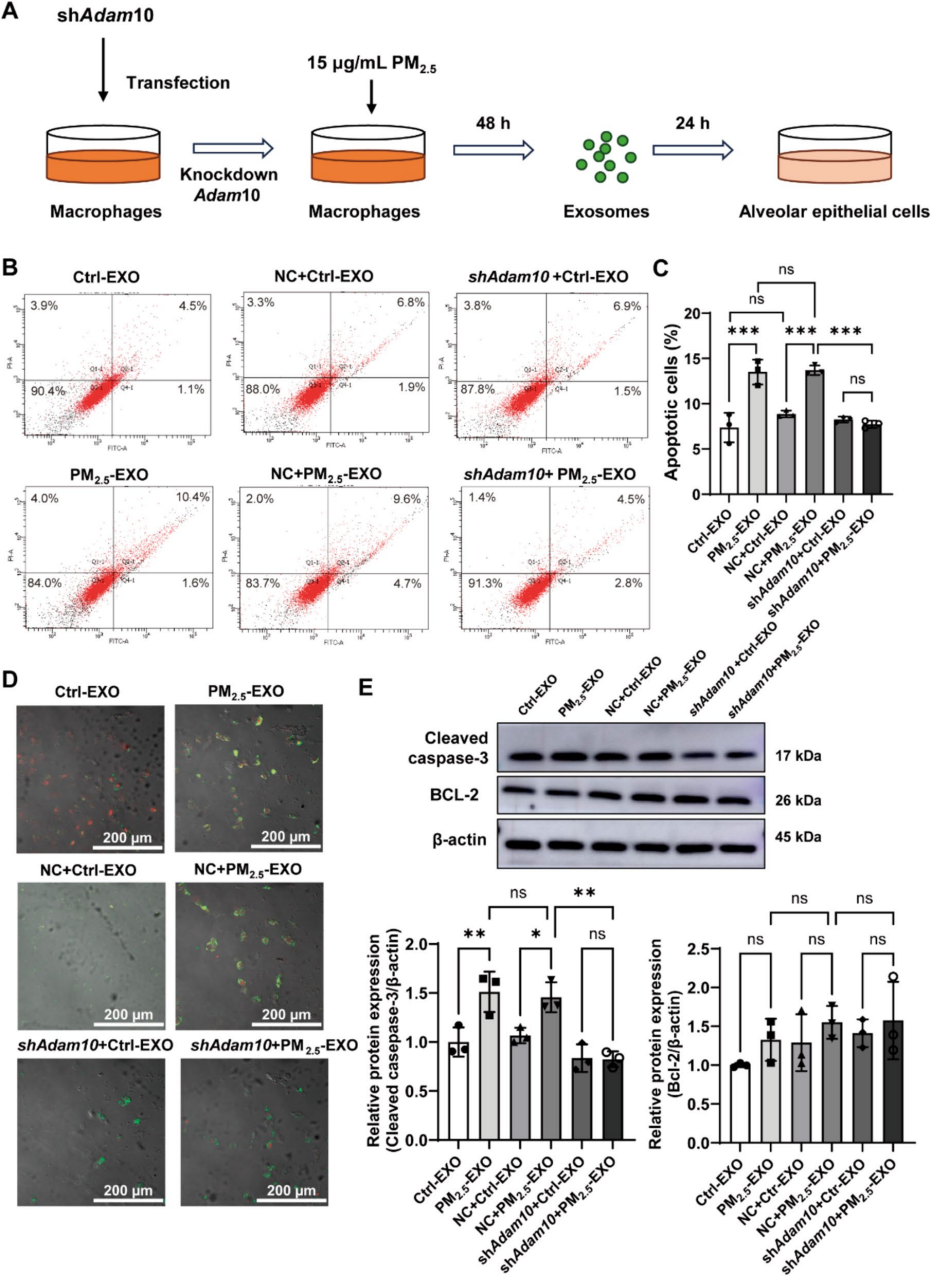
ADAM10 mediates apoptosis in alveolar epithelial cells induced by macrophage-derived exosomes. (**A**) Schematic representation of MLE-12 cells incubated with macrophage-derived exosomes following *Adam10* knockdown.. (**B**, **C**) Flow cytometric analysis of apoptosis in MLE-12 cells using Annexin V-FITC/PI after treatment with exosomes derived from macrophages with or without *Adam10* knockdown. (**D**) Confocal microscopy images of MLE-12 cell apoptosis after treatment with PM_2.5_-exposed macrophage-derived exosomes, with or without *Adam10* knockdown (Annexin V-FITC/PI staining; scale bar = 200 μm). (**E**) Western blot analysis of BCL-2 and cleaved caspase-3 in epithelial cells treated with exosomes from PM_2.5_-stimulated macrophages, with or without *Adam10* knockdown. Group: Ctrl-EXO, exosomes from PBS-stimulated macrophages; PM_2.5_-EXO, exosomes from PM_2.5_-stimulated macrophages; NC, macrophages transduced with non-targeting shRNA; *shAdam10*, macrophages transduced with *shAdam10*-targeting shRNA. Statistical analysis was performed using two-way ANOVA with Tukey’s post hoc test was used to assess the effects of PM_2.5_ and *Adam10* knockdown. Pairwise comparisons were performed using unpaired two-tailed Student’s t-test. Data are presented as mean ± SD; ns, not significant; *p < 0.05, **p < 0.01, ***p < 0.001

Functionally, exosomes from PM_2.5_-stimulated macrophages induced epithelial apoptosis, whereas exosomes from ADAM10-deficient macrophages produced significantly less apoptosis (Fig. S4B, Fig. 6A–D). Cleaved caspase-3, but not BCL-2, increased after PM_2.5_-EXO treatment and decreased with ADAM10 knockdown (Fig. 6E). These findings identify exosomal ADAM10 as an important contributor to the pro-apoptotic activity of macrophage-derived exosomes in this system. These results collectively establish exosomal ADAM10 as a key effector of epithelial apoptosis triggered by PM_2.5_-exposed macrophages, bridging environmental exposure with epithelial cell injury in the pathogenesis of emphysema.

## Discussion

This study identifies an exosome-mediated mechanism by which PM_2.5_, particularly from WS, promotes alveolar epithelial apoptosis and contributes to emphysematous injury. Beyond the well-established roles of inflammation and oxidative stress, our findings reveal that macrophage-derived exosomes serve as active carriers of apoptotic signals and represent a previously underappreciated route of particulate-induced lung damage. PM_2.5_ markedly enhances exosome secretion from macrophages, and these vesicles deliver bioactive ADAM10 to epithelial cells, triggering caspase-3–dependent apoptosis. These results shift the conceptual framework of PM_2.5_ toxicity from a primarily cell-autonomous process to one that involves intercellular propagation of injury via extracellular vesicles.

PM_2.5_ is known to induce lung injury through several classical pathways, particularly inflammation and oxidative stress (Xu et al. 2024; Li et al. 2023). Upon inhalation, PM_2.5_ activates Toll-like receptors (TLRs) on resident lung cells, including macrophages, and epithelial cells, triggering an inflammatory cascade (Brusselle et al. 2011; Takimoto-Sato et al. 2023). This response promotes cytokine and chemokine release, amplifying tissue damage and driving structural changes such as alveolar wall destruction and impaired gas exchange (Zhao et al. 2019). While these mechanisms provide a foundation for understanding PM_2.5_-induced injury, they may not entirely account for the sustained and systemic nature of tissue damage observed in chronic lung diseases. Our study extends this framework by positioning macrophage-derived exosomes as key amplifiers of PM_2.5_ toxicity, providing a mechanism by which particulate-induced signals propagate beyond the initially exposed cells. Importantly, we identify SNAP23 as a critical regulator of this amplification process, demonstrating that PM_2.5_ actively upregulates the SNARE-mediated exosome secretion machinery to enhance the output of pro-injury vesicles.

Alveolar macrophages, the lung's resident immune sentinels, are recognized as primary sources of proteases involved in the pathogenesis of emphysema (Brusselle et al. 2011; Takimoto-Sato et al. 2023; Kapellos et al. 2018), with their accumulation in diseased lungs directly correlating with disease progression and tissue destruction (Gharib et al. 2018). Recent evidence further suggests that macrophages may drive disease progression through noncanonical mechanisms such as the release of exosomes (Kumar and Deep 2020; Neri et al. 2022). These vesicles can carry active enzymes; for example, neutrophil-derived exosomes enriched in neutrophil elastase have been shown to evade α1-antitrypsin inhibition and directly promote extracellular matrix degradation in COPD (Genschmer et al. 2019). Although forced overexpression of ADAM10 in the lung induces emphysema in mice (Saitoh et al. 2009), the mechanistic basis for its pathogenic effect has remained unclear. Apoptosis of alveolar epithelial cells is a key driver of emphysema progression and has been consistently observed in COPD patients and experimental models (Lin et al. 2019; Gu et al. 2015; Chen et al. 2022), we therefore hypothesized that ADAM10-positive exosomes may constitute a functional link.

Our study identifies ADAM10 as a functionally active exosomal cargo, whose abundance increases following PM_2.5_ exposure. Exosomal ADAM10 retains enzymatic activity and directly induces apoptosis in alveolar epithelial cells, establishing a mechanistic connection between particulate exposure, vesicle trafficking, and epithelial injury. Although ADAM10 localizes mainly to the Golgi under basal conditions, increasing evidence supports its presence and enzymatic activity within extracellular vesicles (Gutwein et al. 2003; Kalra et al. 2016; Kowal et al. 2016), where it can cleave substrates such as CD44, L1, and Notch, modulating inflammation and apoptosis (Aljohmani and Yildiz 2020; Lambrecht et al. 2018; Chastagner et al. 2017; Maretzky et al. 2005; Drexhage et al. 2024). ADAM10 also degrades type IV collagen (Saitoh et al. 2009), suggesting potential roles in matrix remodeling. Crucially, the PM_2.5_-induced increase in exosomal ADAM10 is not merely a passive consequence of enhanced exosome production; our SNAP23 knockdown experiments demonstrate that the two processes are mechanistically linked. By upregulating SNAP23, PM2.5 increases the overall output of ADAM10-enriched exosomes, thereby amplifying the pro-apoptotic signal delivered to epithelial cells. This positions SNAP23 and ADAM10 as sequential components of a coordinated pathogenic axis. While the precise upstream apoptotic pathway remains to be fully elucidated, the lack of change in BCL-2 levels suggests that the intrinsic mitochondrial pathway may not be the primary driver. Caspase-3 activation is a common downstream effector in both intrinsic and extrinsic apoptosis (Kumar et al. 2005). It is plausible that exosomal ADAM10 triggers the extrinsic pathway, as ADAM10 is known to modulate death receptor signaling by cleaving substrates such as Fas ligand (Schulte et al. 2007; Kumar et al. 2005). Further investigation is required to delineate the involvement of caspase-8 and the death receptor pathway in this context.

To determine whether PM_2.5_ also modulates the upstream machinery governing exosome release, we examined the SNARE fusion system. Exosome secretion requires fusion of multivesicular bodies with the plasma membrane, a process executed by the conserved SNARE complex. SNAP23, an essential t-SNARE, is broadly required for vesicle–plasma membrane fusion (Liu et al. 2023). Consistent with this role, PM_2.5_ exposure increased SNAP23 protein levels in macrophages, aligning with SNARE-related pathway enrichment in our proteomic dataset. Functionally, SNAP23 knockdown markedly reduced exosome output after PM_2.5_ stimulation, suggesting that particulate exposure enhances vesicle release by engaging core fusion machinery. This finding is conceptually novel, as it identifies the exosome secretion machinery itself, rather than just exosome cargo composition, as a target of PM_2.5_ toxicity. Although previous studies have documented PM_2.5_-induced changes in exosomal miRNA or protein profiles, our work reveals an additional layer of regulation at the level of exosome biogenesis and release. While this may influence the abundance of ADAM10-positive vesicles, it remains uncertain whether PM_2.5_ alters ADAM10 loading per vesicle or simply increases vesicle number. Although immuno-EM revealed higher ADAM10 labeling density on PM_2.5_-derived exosomes, its semi-quantitative nature limits definitive interpretation.

Beyond its local effects in the lung, PM_2.5_ may also contribute to systemic health impacts through the release of exosomes from activated lung macrophages, which could propagate injury signals to distant organs, thereby promoting systemic inflammation and contributing to extrapulmonary diseases such as cardiovascular events (Eckhardt et al. 2022; Gao et al. 2025). Conversely, circulating exosomes from other tissues could also be altered by PM_2.5_-induced systemic inflammation, potentially creating a complex interplay between pulmonary and systemic responses. This represents an important direction for future research. Analyzing plasma/serum exosomes could, on one hand, serve as non-invasive biomarkers for monitoring PM_2.5_-induced lung injury, particularly to determine whether the SNAP23–ADAM10 axis identified in our pulmonary model is detectable in the circulation and correlates with disease severity. On the other hand, these circulating vesicles may act as potential mediators of the systemic health effects of air pollution, thereby extending our mechanistic findings from the lung to extrapulmonary tissues.

Our study provides compelling evidence that PM_2.5_ exposure promotes emphysema progression through enhanced macrophage exosome secretion and ADAM10-mediated alveolar epithelial apoptosis. The exosomal ADAM10 pathway represents an important, but not exclusive, driver of PM_2.5_-induced epithelial injury; additional mechanisms—including oxidative stress, inflammatory signaling, and direct particulate toxicity—are also likely to contribute. Furthermore, our current work focused primarily on PM_2.5_ and did not fully address potential interactions with coexisting wood smoke pollutants such as VOCs, PAHs, and NOx. Future studies should adopt a multifaceted framework to investigate the combined effects of complex pollutant mixtures on alveolar injury. The presence of CD63-ADAM10 co-localization in lungs of COPD patients, whose disease etiology is multifactorial and includes smoking, suggests that this exosome-mediated pathway may represent a common pathological mechanism of alveolar destruction, irrespective of the initial injurious agent. This convergence supports the broader relevance of our findings beyond wood smoke-specific exposure. Although our mechanistic findings were supported by increased ADAM10 expression in lung tissues from COPD patients, expanded clinical analyses across diverse patient cohorts will be necessary to determine the overall contribution of this pathway to human disease progression. It remains unclear whether PM_2.5_ alters circulating exosomes in blood, and future work could explore whether the SNAP23–ADAM10 axis is detectable in plasma and correlates with disease severity.

A limitation of our *in vivo* study is the use of GW4869, a pharmacological inhibitor that blocks exosome generation from all cell types by inhibiting neutral sphingomyelinase, an enzyme involved in ceramide-mediated exosome biogenesis. Therefore, while our in vitro experiments with macrophage cultures demonstrate that PM2.5 enhances exosome release from macrophages (Fig. 2D-H), and our rescue studies using intratracheal administration of macrophage-derived exosomes (Fig. 2I-J) show that these vesicles are sufficient to induce emphysematous changes, the protective effect of GW4869 *in vivo* could also involve inhibition of exosome release from other lung cells, such as epithelial cells, endothelial cells, or fibroblasts. This is an inherent limitation of pharmacological inhibition studies and prevents us from definitively concluding that macrophages are the exclusive source of pathogenic exosomes *in vivo*. Future studies using macrophage-specific genetic models (e.g., LysM-Cre mediated deletion of nSMase2) or adoptive transfer experiments would provide definitive evidence for the specific role of macrophage-derived exosomes in WS-induced emphysema. We also acknowledge that our in vitro studies used murine cell lines (MLE-12 and RAW264.7), while *in vivo* validation was performed in rats. This cross-species approach was necessitated by practical advantages of the rat model for chronic inhalation studies. However, given the high conservation of apoptotic pathways and ADAM10 signaling across mammals, the convergence of findings from both models supports the validity of our conclusions. Future studies using fully homologous rat primary cell systems would further strengthen this validation.

## Conclusion

This study identifies macrophage–epithelial exosomal communication as a driver of PM_2.5_-induced emphysematous injury. Through integrated cellular and molecular analyses, we show that particulate exposure reprograms macrophage vesicle trafficking and enhances exosomal delivery of the pro-injury sheddase ADAM10 to alveolar epithelial cells. These findings broaden current understanding of how biomass-derived particulates disrupt lung homeostasis and highlight exosome-associated proteases as potential intervention targets. As biomass burning and wildfire events continue to intensify globally, delineating these intercellular pathways provides a foundation for strategies aimed at reducing smoke-related lung damage.

## Supplementary Information

Below is the link to the electronic supplementary material.

Supplementary file1 (PDF 267 KB)

Supplementary file2 (DOCX 823 KB)

## Data Availability

Raw data for this study were generated at Guangzhou Medical University, Guangzhou, China. The datasets generated and/or analyzed during the current study are available from the corresponding author upon reasonable request.
