# Supplementary material for: Macrophage exosomal ADAM10 mediates alveolar epithelial apoptosis induced by wood smoke PM2.5
Source: Cell Biol Toxicol. 2026 Mar 29;42(1):60. doi: 10.1007/s10565-026-10179-y (PMC13156083; doi:10.1007/s10565-026-10179-y)
Supplement: Supplementary file 1 — Supplementary file1 (PDF 267 KB) [file 10565_2026_10179_MOESM1_ESM.pdf]

Electrophoresis & Brownian Motion
Video Analysis
Laser Scattering Microscopy

www.particle-metrix.de

Operator (Report): PMX
Video Operator: PMX

Sample Parameters
Sample Name: cell-control
Comment: Sample Remarks0:
Sample Remarks1:
Sample Remarks2:
Electrolyte: PBS
Temperature: 24.13 □ sensed
pH 7.4 entered
Conductivity: 15000.00 □/cm entered

Measurement Parameters
Cell S/N: NTA-0389

Measurement Mode: Size Distribution 2 Cycles
11 Positions, 2 Removed for Analysis

Result (sizes in nm)

	Number	Concentration	Volume
Median (X50)	166.1	166.1	265.7
Span	74.5	74.5	101.7

Concentration: 8.0E+6 Particles / mL
Dilution Factor: 35
Original Concentration: 2.8E+8 Particles / mL

Quality
Average Counted Particles per Frame: 22
Number of Traced Particles: 179

Analysis Parameters
Max Area: 1000, Min Area: 20, Min Brightness: 20

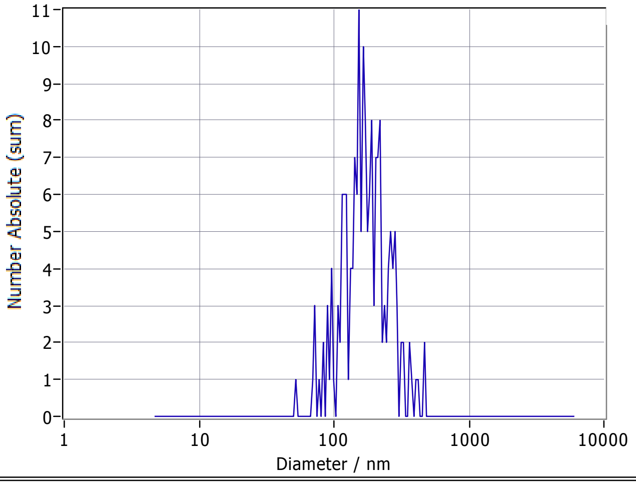

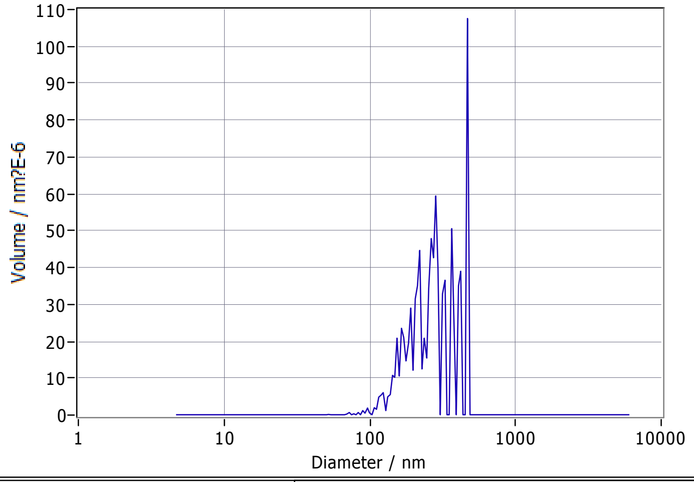

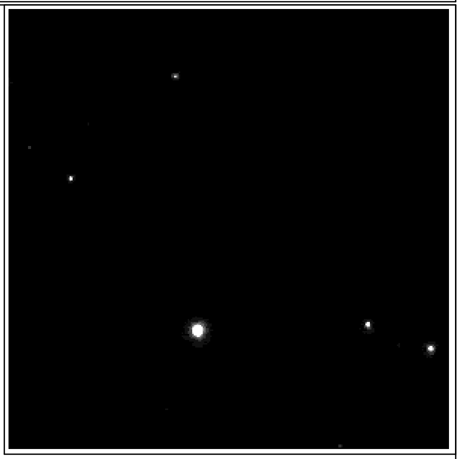

Peak Analysis (Number Absolute)

Diameter / nm	Number Absolute	FWHM / nm	Percentage
161.2	8.3	84.8	57.5
119.5	6.3	12.1	12.9
265.5	5.1	46.5	19.5
96.4	2.4	15.9	5.1
72.4	1.7	7.5	5.0

X Values

	Number	Concentration	Volume
X10	104.5	104.5	160.0
X50	166.1	166.1	265.7
X90	275.2	275.2	454.2
Span	1.0	1.0	1.1
Mean	184.6	184.6	285.0
StdDev	74.5	74.5	101.7

Comment

(Signature)

Analyzed Video: D:\ZMJ\2025-03-05\20250305_0002_cell-control_size.avi

ZetaVIEW S/N 18-385, Software ZetaView 8.04.02 SP2, Camera 0.714 □/px

Experiment: 2025-03-05 14:37, Report: 2025-03-05 14:39

Electrophoresis & Brownian Motion
Video Analysis
Laser Scattering Microscopy

www.particle-metrix.de

Operator (Report): PMX

Video Operator: PMX

Sample Parameters

Sample Name: CELL-CONTROL
Comment: Sample Remarks0:
Sample Remarks1:
Sample Remarks2:
Electrolyte: PBS
Temperature: 23.52 □ sensed
pH 7.4 entered
Conductivity: 15000.00 □/cm entered

Result (sizes in nm)

	Number	Concentration	Volume
Median (X50)	0.0	0.0	0.0
Span			

Concentration: NaN Particles / mL
Dilution Factor: 500
Original Concentration: NaN Particles / mL

Quality

Average Counted Particles per Frame: 2147483647
Number of Traced Particles: 0

Measurement Parameters

Cell S/N: NTA-0389

Measurement Mode: Size Distribution 2 Cycles

11 Positions, 11 Removed for Analysis

Analysis Parameters

Max Area: 1000, Min Area: 20, Min Brightness: 20

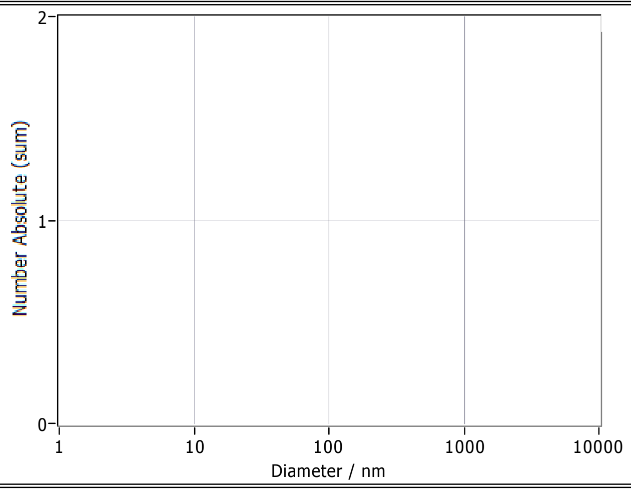

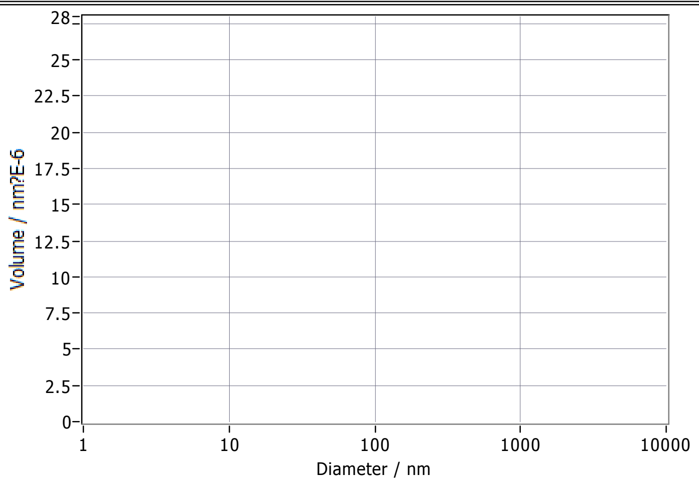

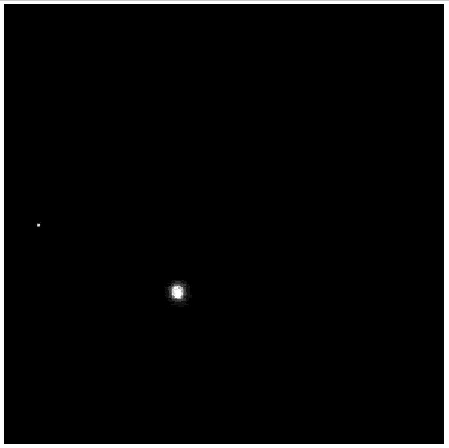

Peak Analysis (Number Absolute)

Diameter / nm	Number Absolute	FWHM / nm	Percentage

X Values

	Number	Concentration	Volume
X10			
X50			
X90			
Span			
Mean			
StdDev			

Comment

(Signature)

Analyzed Video: D:\ZMJ\2025-04-10\20250410_0001_CELL-CONTROL_size.avi

ZetaVIEW S/N 18-385, Software ZetaView 8.04.02 SP2, Camera 0.714 □/px

Experiment: 2025-04-10 14:23, Report: 2025-04-10 14:26

Electrophoresis & Brownian Motion
Video Analysis
Laser Scattering Microscopy

www.particle-metrix.de

Operator (Report): PMX

Video Operator: PMX

Sample Parameters

Sample Name: CELL-CONTROL-3
Comment: Sample Remarks0:
Sample Remarks1:
Sample Remarks2:
Electrolyte: PBS
Temperature: 23.76 □ sensed
pH 7.4 entered
Conductivity: 15000.00 □/cm entered

Result (sizes in nm)

	Number	Concentration	Volume
Median (X50)	0.0	0.0	0.0
Span			

Concentration:	NaN Particles / mL
Dilution Factor:	500
Original Concentration:	NaN Particles / mL

Quality

Average Counted Particles per Frame: 2147483647
Number of Traced Particles: 0

Measurement Parameters

Cell S/N: NTA-0389

Measurement Mode: Size Distribution 2 Cycles

11 Positions, 11 Removed for Analysis

Analysis Parameters

Max Area: 1000, Min Area: 20, Min Brightness: 20

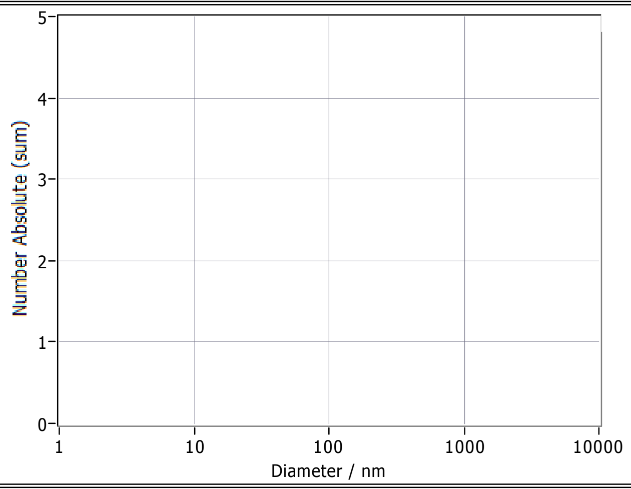

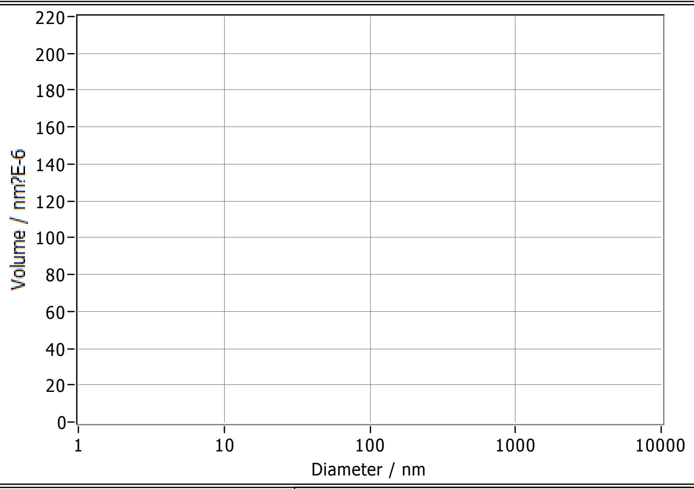

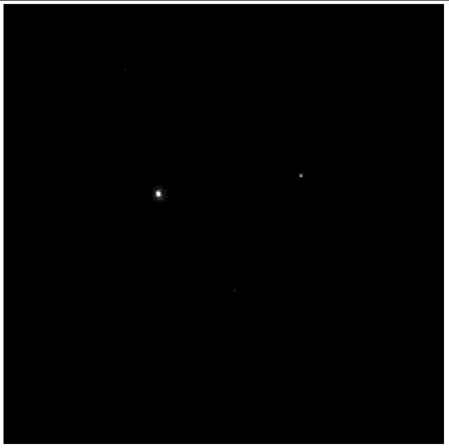

Peak Analysis (Number Absolute)

Diameter / nm	Number Absolute	FWHM / nm	Percentage

X Values

	Number	Concentration	Volume
X10			
X50			
X90			
Span			
Mean			
StdDev			

Comment

(Signature)

Analyzed Video: D:\ZMJ\2025-04-10\20250410_0003_CELL-CONTROL-3_size.avi

ZetaVIEW S/N 18-385, Software ZetaView 8.04.02 SP2, Camera 0.714 □/px

Experiment: 2025-04-10 14:31, Report: 2025-04-10 14:34

ZetaView
PARTICLE METRIX
www.particle-metrix.de

Electrophoresis & Brownian Motion Video Analysis Laser Scattering Microscopy

Operator (Report): PMX
Video Operator: PMX

Sample Parameters
Sample Name: cell-PM2.5
Comment: Sample Remarks0:
Sample Remarks1:
Sample Remarks2:
Electrolyte: PBS
Temperature: 23.97 □ sensed
pH 7.4 entered
Conductivity: 15000.00 □/cm entered

Result (sizes in nm)

	Number	Concentration	Volume
Median (X50)	145.5	145.5	203.9
Span	56.0	56.0	62.0

Concentration: 5.8E+6 Particles / mL
Dilution Factor: 50
Original Concentration: 2.9E+8 Particles / mL

Quality
Average Counted Particles per Frame: 16
Number of Traced Particles: 106

Measurement Parameters
Cell S/N: NTA-0389

Measurement Mode: Size Distribution 2 Cycles
11 Positions, 3 Removed for Analysis

Analysis Parameters
Max Area: 1000, Min Area: 20, Min Brightness: 20

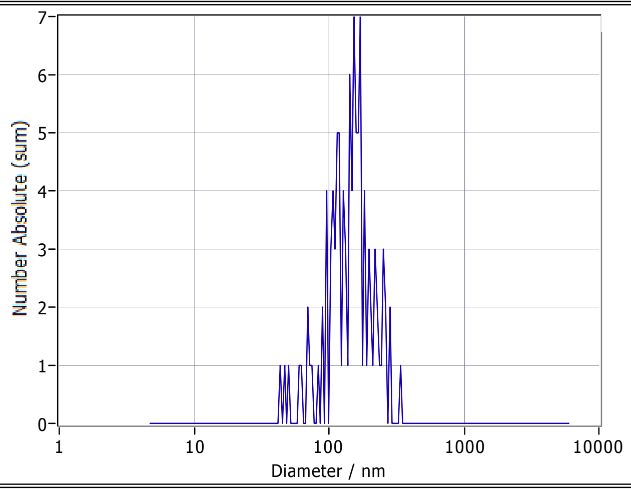

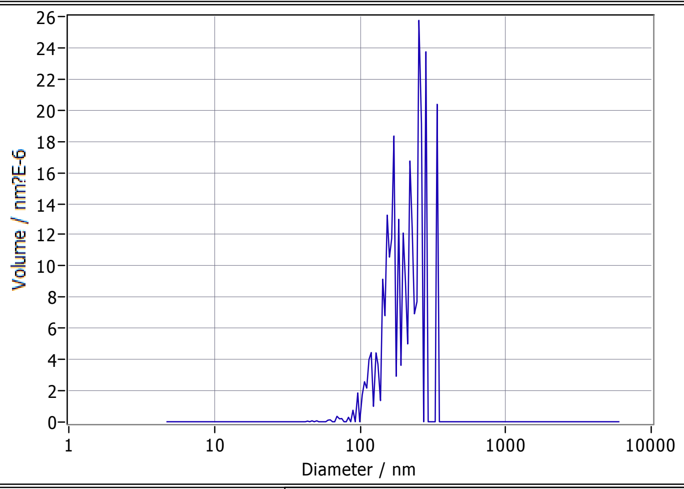

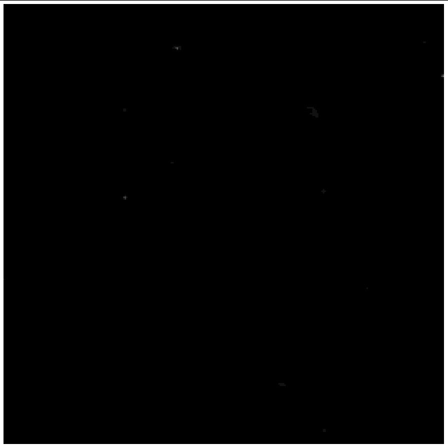

Peak Analysis (Number Absolute)

Diameter / nm	Number Absolute	FWHM / nm	Percentage
158.3	5.9	32.7	47.1
114.1	4.4	20.3	25.7
228.1	1.8	0.0	1.8
243.5	1.5	50.7	14.4
71.4	1.4	6.7	11.1

X Values

	Number	Concentration	Volume
X10	87.3	87.3	132.5
X50	145.5	145.5	203.9
X90	225.7	225.7	279.9
Span	1.0	1.0	0.7
Mean	151.6	151.6	211.1
StdDev	56.0	56.0	62.0

Comment

(Signature)

Analyzed Video: D:\ZMJ\2025-03-05\20250305_0003_cell-PM2.5_size.avi

ZetaVIEW S/N 18-385, Software ZetaView 8.04.02 SP2, Camera 0.714 □/px

Experiment: 2025-03-05 14:43, Report: 2025-03-05 14:46

Electrophoresis & Brownian Motion
Video Analysis
Laser Scattering Microscopy

ZetaVIEW® Laser Scattering Video Microscope
PARTICLEMETRIX
www.particle-metrix.de

Operator (Report): PMX
Video Operator: PMX

Sample Parameters
Sample Name: CELL-PM2.5
Comment: Sample Remarks0:
Sample Remarks1:
Sample Remarks2:
Electrolyte: PBS
Temperature: 23.89 □ sensed
pH 7.4 entered
Conductivity: 15000.00 □/cm entered

Result (sizes in nm)

	Number	Concentration	Volume
Median (X50)	0.0	0.0	0.0
Span			

Concentration: NaN Particles / mL
Dilution Factor: 500
Original Concentration: NaN Particles / mL

Quality
Average Counted Particles per Frame: 2147483647
Number of Traced Particles: 0

Measurement Parameters
Cell S/N: NTA-0389

Measurement Mode: Size Distribution 2 Cycles
11 Positions, 11 Removed for Analysis

Analysis Parameters
Max Area: 1000, Min Area: 20, Min Brightness: 20

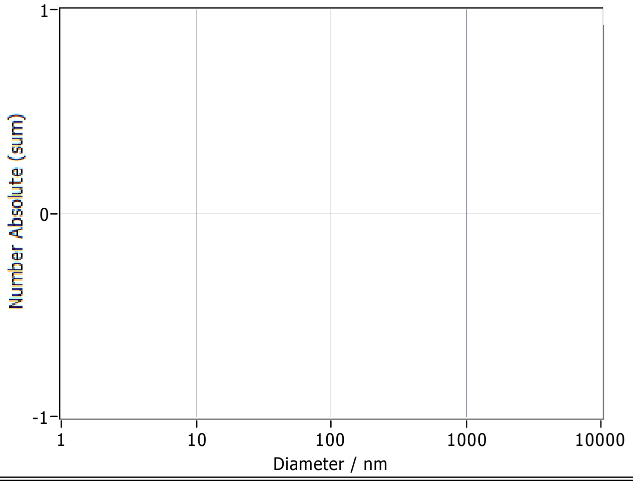

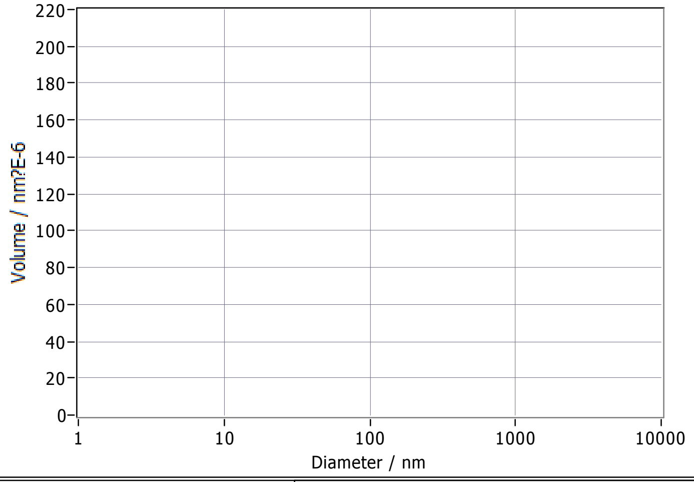

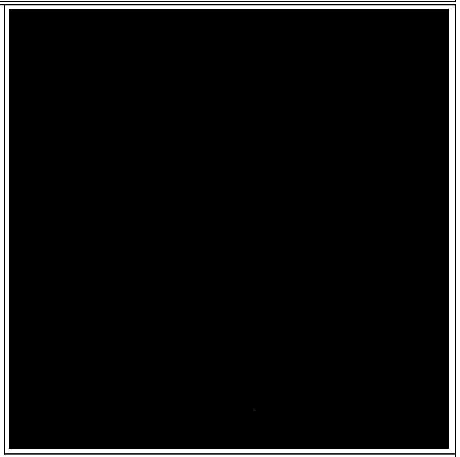

Peak Analysis (Number Absolute)

Diameter / nm	Number Absolute	FWHM / nm	Percentage

X Values

	Number	Concentration	Volume
X10			
X50			
X90			
Span			
Mean			
StdDev			

Comment

(Signature)

Analyzed Video: D:\ZMJ\2025-04-10\20250410_0004_CELL-PM2.5_size.avi

ZetaVIEW S/N 18-385, Software ZetaView 8.04.02 SP2, Camera 0.714 □/px

Experiment: 2025-04-10 14:36, Report: 2025-04-10 14:38

Electrophoresis & Brownian Motion
Video Analysis
Laser Scattering Microscopy

Operator (Report): PMX
Video Operator: PMX

Sample Parameters
Sample Name: CELL-PM2.5-2
Comment: Sample Remarks0:
Sample Remarks1:
Sample Remarks2:
Electrolyte: PBS
Temperature: 23.94 □ sensed
pH 7.4 entered
Conductivity: 15000.00 □/cm entered

Result (sizes in nm)

	Number	Concentration	Volume
Median (X50)	0.0	0.0	0.0
Span			

Concentration: NaN Particles / mL
Dilution Factor: 500
Original Concentration: NaN Particles / mL

Quality
Average Counted Particles per Frame: 2147483647
Number of Traced Particles: 0

Measurement Parameters
Cell S/N: NTA-0389

Measurement Mode: Size Distribution 2 Cycles
11 Positions, 11 Removed for Analysis

Analysis Parameters
Max Area: 1000, Min Area: 20, Min Brightness: 20

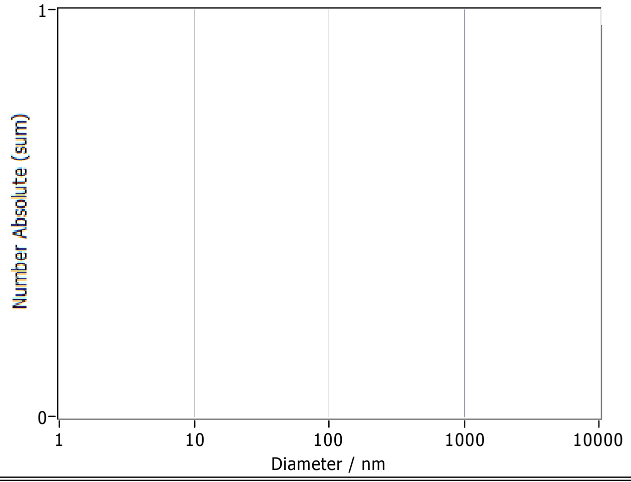

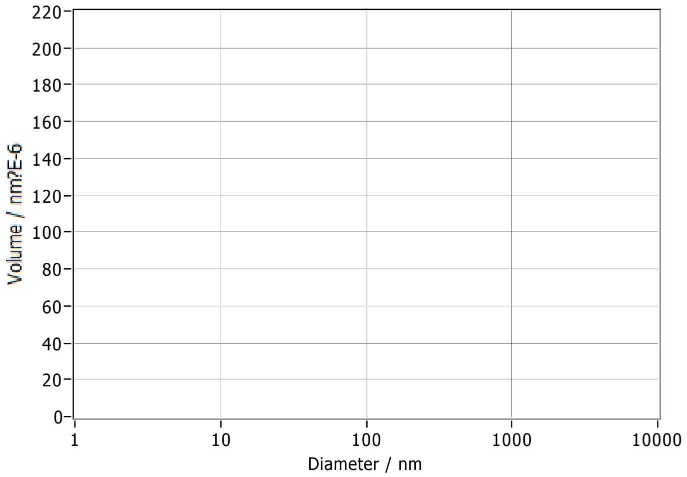

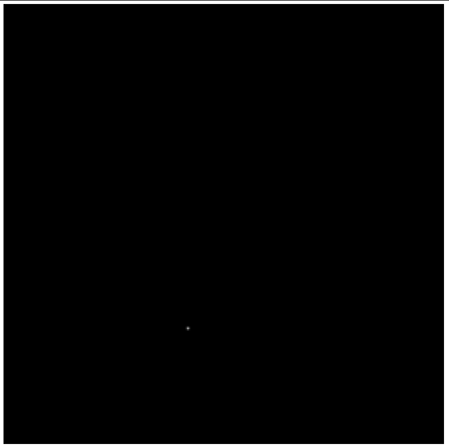

Peak Analysis (Number Absolute)

Diameter / nm	Number Absolute	FWHM / nm	Percentage

X Values

	Number	Concentration	Volume
X10			
X50			
X90			
Span			
Mean			
StdDev			

Comment

(Signature)

Analyzed Video: D:\ZMJ\2025-04-10\20250410_0005_CELL-PM2.5-2_size.avi

ZetaVIEW S/N 18-385, Software ZetaView 8.04.02 SP2, Camera 0.714 □/px

Experiment: 2025-04-10 14:39, Report: 2025-04-10 14:41
