# Supplementary material for: Macrophage exosomal ADAM10 mediates alveolar epithelial apoptosis induced by wood smoke PM2.5
Source: Cell Biol Toxicol. 2026 Mar 29;42(1):60. doi: 10.1007/s10565-026-10179-y (PMC13156083; doi:10.1007/s10565-026-10179-y)
Supplement: Supplementary file 2 — Supplementary file2 (DOCX 823 KB) [file 10565_2026_10179_MOESM2_ESM.docx]

**Table S1. Summary of PM_2.5_ Exposure Scenarios and Cumulative Daily Exposure Levels**

| Exposure scenario | PM_2.5_  (mg/m³) | Daily exposure duration(h) | Cumulative exposure  (mg·h/m³/day) | Notes |
| --- | --- | --- | --- | --- |
| Rat experiment | 15 | 6 | 90.0 |  |
| General population (urban air) | 0.015 | 24 | 0.36 | Representing the WHO 24-hour guideline level (15 μg/m³) |
| Biomass fuel users (high pollution) | traditional stove: 0.408  improved stove: 0.205 | 24 | 9.6  4.8 | The average 24-hour indoor PM_2.5_ concentration in rural homes is 408 μg/m³ with traditional stoves and 205 μg/m³ with improved stoves [29] |

**Table S2. Summary of Clinical Information for Lung Tissue Donors**

| Group | ID (Last Digits) | Sex | Age | Smoking History (0 = No, 1 = Yes) | GOLD Stage | Notes |
| --- | --- | --- | --- | --- | --- | --- |
| Ctrl | 29 | F | 71 | 0 | 0 |  |
| Ctrl | 43 | M | 73 | 1 | 0 |  |
| Ctrl | 47 | M | 50 | 0 | 0 |  |
| COPD | 8 | M | 77 | 0 | 2 |  |
| COPD | 12 | F | 56 | 0 | 1 |  |
| COPD | 48 | M | 73 | 1 | 2 |  |

**Table S3. Proteomic Analysis of Exosomes Derived from PM_2.5_-Stimulated Macrophages: Proteins Involved in SNAP Receptor Activity and SNARE Interactions in Vesicular Transport.**

| **Protein accession** | **Protein description** | **Gene name** | **Mol. weight [kDa]** | **P/C Ratio** | **P value** |
| --- | --- | --- | --- | --- | --- |
| P70452 | Syntaxin-4 | Stx4 | 34.165 | 1.681 | 1.31E-02 |
| O70439 | Syntaxin-7 | Stx7 | 29.82 | 1.546 | 1.80E-02 |
| O88983 | Syntaxin-8 | Stx8 | 26.925 | 1.517 | 1.05E-02 |
| Q9ER00 | Syntaxin-12 | Stx12 | 31.195 | 1.962 | 4.58E-03 |
| O70480 | Vesicle-associated membrane protein 4 | Vamp4 | 16.353 | 1.778 | 2.08E-02 |
| O70404 | Vesicle-associated membrane protein 8 | Vamp8 | 11.451 | 1.754 | 1.94E-02 |
| O09044 | Synaptosomal-associated protein 23 | Snap23 | 23.261 | 1.669 | 3.46E-02 |
| O88384 | Vesicle transport through interaction with t-SNAREs homolog 1B | Vti1b | 26.713 | 1.611 | 3.01E-02 |


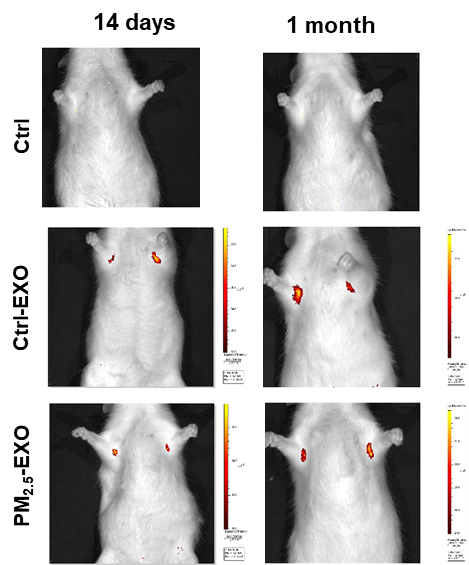


**Figure S1. In vivo localization of PKH67-labeled macrophage-derived exosomes after intratracheal instillation in rats.** Small-animal fluorescence imaging was performed at 14 days and 1 month following intratracheal administration of PKH67-labeled exosomes. Rats were assigned to three groups: Ctrl (no exosome treatment), Ctrl-EXO (exosomes from PBS-treated RAW264.7 macrophages), and PM_2.5_-EXO (exosomes from PM_2.5_-stimulated macrophages). Fluorescent signals indicate the biodistribution and retention of exosomes in vivo over time.


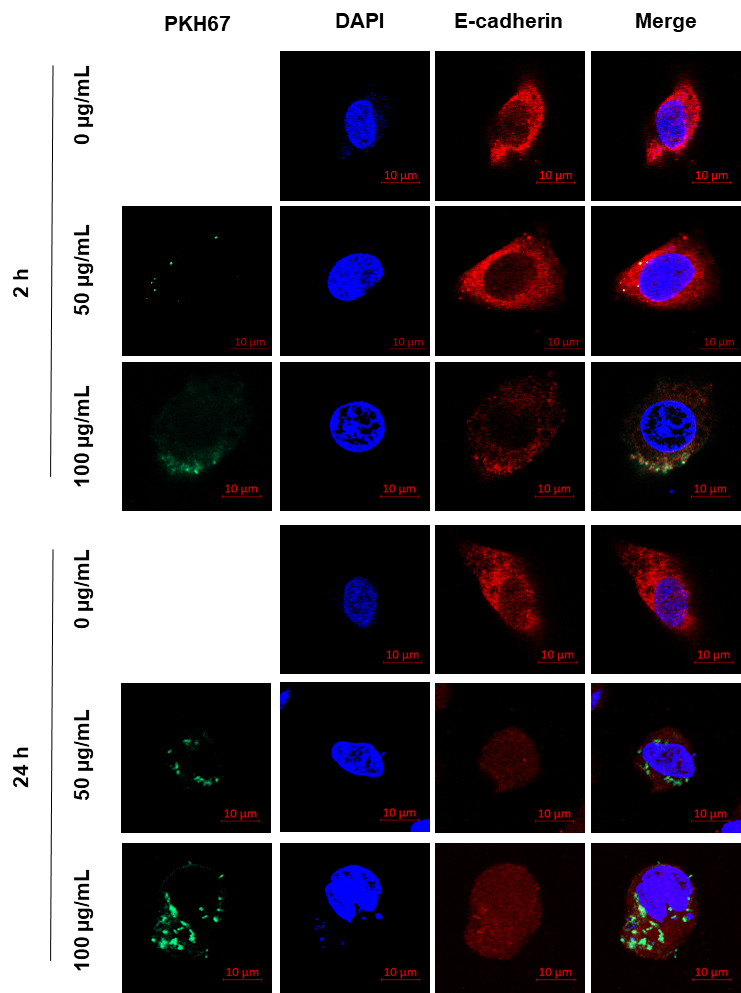


**Figure S2.** **Fluorescent microscopy images of MLE12 cells incubated with PKH67-labeled exosomes from PBS-treated macrophages.** Cells were treated with exosomes at concentrations of 50 μg/mL and 100 μg/mL for either 2 hours or 24 hours (scale bar = 10 µm).


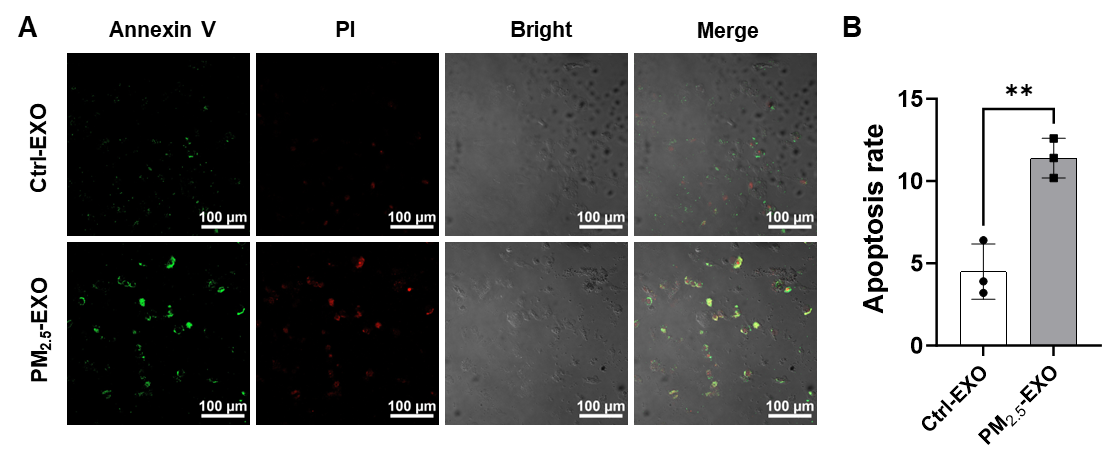


**Figure S3.** **PM_2.5_-stimulated macrophage-derived exosomes promote apoptosis in epithelial cells.** (A) Confocal microscopy images showing the apoptosis of MLE12 cells induced by PM_2.5_-treated macrophage exosomes, detected by annexin V FITC/PI staining. (B) Quantitative analysis of apoptosis in MLE12 cells (n=3). Data are presented as mean ± SD. Statistical significance is indicated as **p<0.01; scale bar = 100 µm.

**
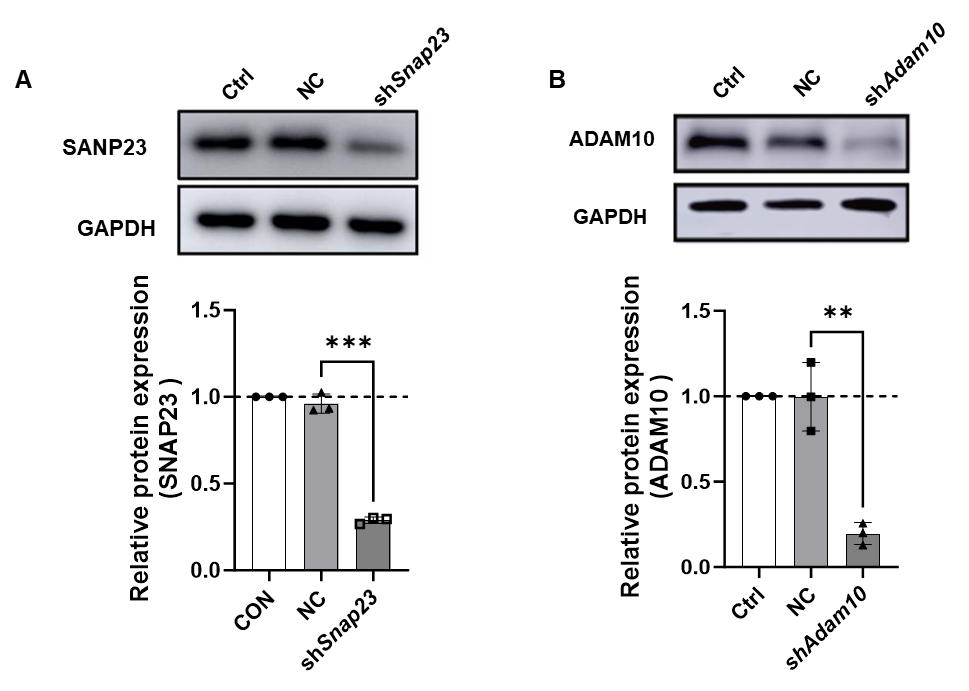
**

**Figure S4. Validation of *Snap23* and *Adam10* knockdown efficiency in macrophages.** (A) Western blot analysis demonstrating successful knockdown of SNAP23 protein expression in macrophages following shRNA transfection. (B) Western blot analysis confirming reduced Adam10 protein levels after shRNA-mediated knockdown. Ctrl: Untreated macrophages. NC: Negative control vector-transfected macrophages treated with PBS; sh*Snap23*: Macrophages transfected with *Snap23*-targeting shRNA; sh*Adam10*: Macrophages transfected with *Adam10*-targeting shRNA. Data represent mean ± SD of three independent experiments. Statistical significance was determined by Student's t-test (**p < 0.01, ***p < 0.001 vs NC group)
